# Performance of an electronic health record-based predictive model to identify patients with atrial fibrillation across countries

**DOI:** 10.1371/journal.pone.0269867

**Published:** 2022-07-08

**Authors:** Ruth Mokgokong, Renate Schnabel, Henning Witt, Robert Miller, Theodore C. Lee

**Affiliations:** 1 Health Economics & Outcomes Research, Internal Medicine, Pfizer, Surrey, United Kingdom; 2 University Heart & Vascular Center Hamburg Eppendorf, Hamburg, Germany; 3 German Center for Cardiovascular Research (DZHK) Partner Site, Hamburg/Kiel/Lübeck, Germany; 4 Pfizer Germany, Berlin, Germany; 5 Internal Medicine, Pfizer, New York City, New York, United States of America; Albert Einstein College of Medicine, UNITED STATES

## Abstract

**Background:**

Atrial fibrillation (AF) burden on patients and healthcare systems warrants innovative strategies for screening asymptomatic individuals.

**Objective:**

We sought to externally validate a predictive model originally developed in a German population to detect unidentified incident AF utilising real-world primary healthcare databases from countries in Europe and Australia.

**Methods:**

This retrospective cohort study used anonymized, longitudinal patient data from 5 country-level primary care databases, including Australia, Belgium, France, Germany, and the UK. The study eligibility included adult patients (≥45 years) with either an AF diagnosis (cases) or no diagnosis (controls) who had continuous enrolment in the respective database prior to the study period. Logistic regression was fitted to a binary response (yes/no) for AF diagnosis using pre-determined risk factors.

**Results:**

AF patients were from Germany (n = 63,562), the UK (n = 42,652), France (n = 7,213), Australia (n = 2,753), and Belgium (n = 1,371). Cases were more likely to have hypertension or other cardiac conditions than controls in all validation datasets compared to the model development data. The area under the receiver operating characteristic (ROC) curve in the validation datasets ranged from 0.79 (Belgium) to 0.84 (Germany), comparable to the German study model, which had an area under the curve of 0.83. Most validation sets reported similar specificity at approximately 80% sensitivity, ranging from 67% (France) to 71% (United Kingdom). The positive predictive value (PPV) ranged from 2% (Belgium) to 16% (Germany), and the number needed to be screened was 50 in Belgium and 6 in Germany. The prevalence of AF varied widely between these datasets, which may be related to different coding practices. Low prevalence affected PPV, but not sensitivity, specificity, and ROC curves.

**Conclusions:**

AF risk prediction algorithms offer targeted ways to identify patients using electronic health records, which could improve screening number and the cost-effectiveness of AF screening if implemented in clinical practice.

## Introduction

Atrial fibrillation (AF) is a common type of arrhythmia that increases the risk for stroke by 5-fold potentially leading to early mortality [1, 2]. In 2016, the prevalence of AF was 46.3 million worldwide, and has increased roughly three-fold in the last 50 years [3, 4]. Additionally, AF risk level in older men is reportedly higher than in women [5, 6]. The overall prevalence of AF may be higher due to the clinical challenge of diagnosing paroxysmal and/or asymptomatic cases [7]. Timely treatment of AF could reduce the risk of stroke and stroke-related morbidity and mortality [8]; however, many patients are unaware of their condition until after enduring an acute ischemic stroke [9]. This arrhythmia is associated with significant healthcare expenditures primarily due to hospital admissions costs [10].

The burden of AF on patients and on the healthcare system warrants innovative strategies for screening asymptomatic individuals [11]. Mass screening with electrocardiography (ECG) would be expensive and 83 individuals would need to be screened to identify one individual with treatable AF [12]. Therefore, guidelines recommend opportunistic screening during regular health visits in patients >65 years by either ECG, which is the gold standard for diagnosing AF, or by pulse taking, and systematic screening in patients ≥75 years to detect AF [13]. The recommendation for opportunistic screening is designated as Class I (level B for evidence) and the recommendation for systematic screening is designated as Class IIa (level B for evidence) [13].

A more cost-effective and novel approach to pre-select patients for AF screening involves using routinely collected data. Machine learning can be used to develop predictive models to select patients who have specific risk factors and who should be screened for AF using an ECG. Automated machine learning has previously assessed the discriminatory ability of models to screen for AF using large claims databases and electronic health records [14–16]. Predictive tools that can identify risk factors and other predictors for AF and that can be applied to routinely collected data offer a targeted approach leading to screening in the primary care setting [7]. Big data and automated analysis in healthcare provides an avenue for digital innovation and personalized medicine, and further research is needed to evaluate whether predictive modeling effectively identifies AF in different sources of data. The purpose of this study was to externally validate a model developed by Schnabel et al. in a German population [16] for the prediction of incident AF utilising several real-world primary healthcare databases from countries in Europe and in Australia.

## Methods

### Study design

**Fig 1:**
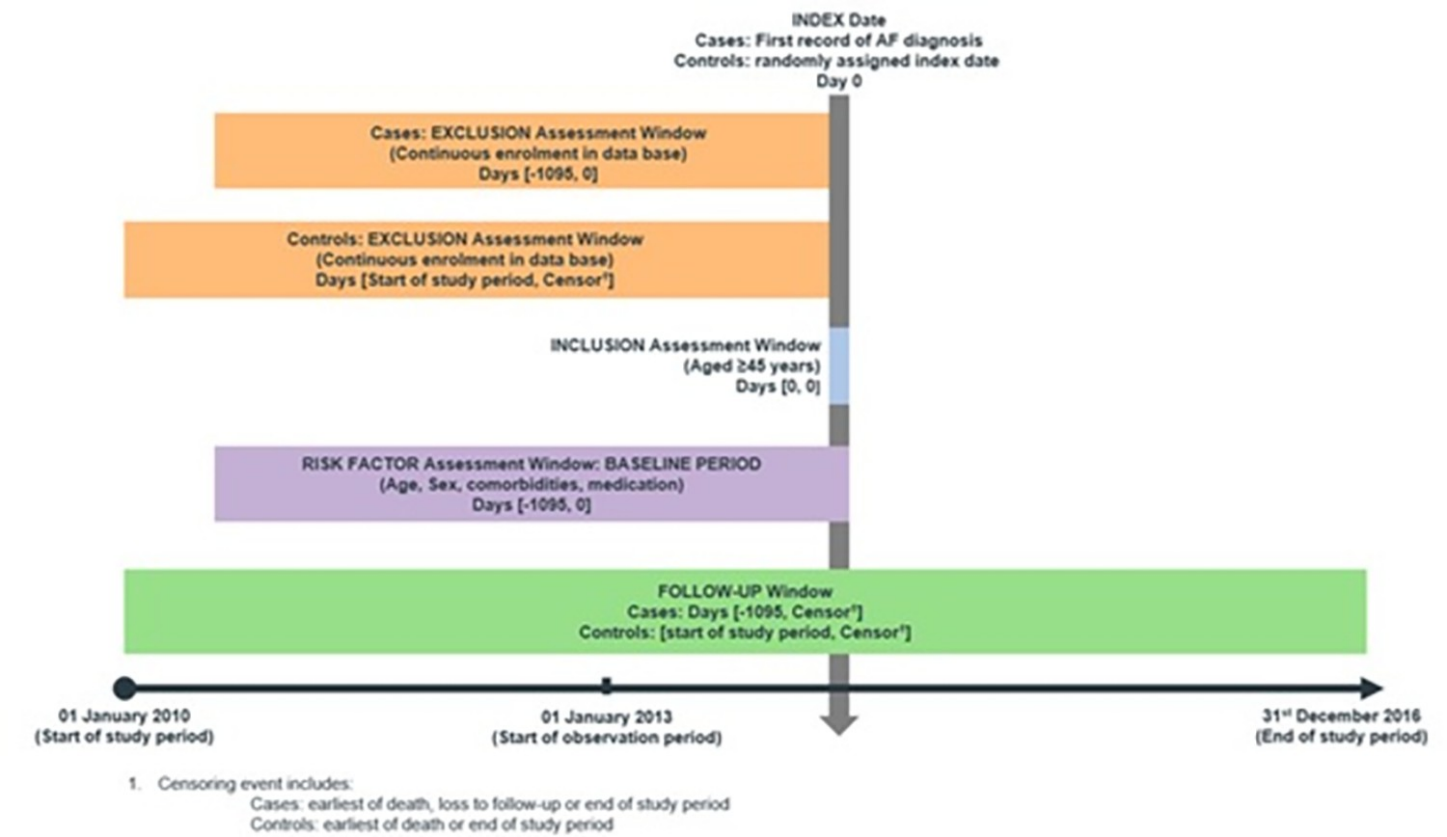
Study design inclusion and exclusion criteria.

This retrospective cohort study used anonymized, longitudinal patient data from 5 country-level IQVIA databases, including Australia, Belgium, France, Germany, and the United Kingdom. The German dataset in this validation study is a primary care electronic medical record and differs from the dataset used in the model development study, which was a claims dataset (**S1 Table in S1 File** for countrywide comparison of datasets). The study eligibility included adult patients (≥45 years) who had continuous enrolment in the respective database prior to the study period (**Fig 1**). Patients were considered to have incident AF if they had an AF International Classification of Diseases– 10^th^ edition (ICD-10) diagnosis code during the observation period. Control patients had no specific AF diagnosis during the entire study. The study was approved by IQVIA IMRD Independent Scientific Review Committee. Patient consent was waived by the ethics committee because data were fully anonymized.

### Observation period

The baseline period was 1,095 days prior to the index date, and this was used to identify eligible cases and assess patient risk factor information for both cases and controls. The observation period for cases was from January 1^st^, 2013 to December 31^st^, 2016. Control patients who were not diagnosed with AF during the study duration were observed from January 1^st^, 2010 to December 31^st^, 2016. All patients had an index date recorded during the observation period. In cases, the index case was the first date of an AF diagnosis, whereas controls were assigned a pseudo index date given they had no AF diagnosis. A sensitivity analysis was performed using the most recent data extracted from the Germany and France database (**S1 Fig in S1 File** for the study design of sensitivity analysis). In the Germany dataset, the study period was shifted to start from January 1^st^, 2013 to December 31^st^, 2019, and the observation period was from January 1^st^, 2016 to December 31^st^, 2019. In the France dataset, the study period was shifted to start from January 1^st^, 2012 to December 31^st^, 2018, and the observation period was from January 1^st^, 2015 to December 31^st^, 2018.

### Covariates

This study described and compared risk factors included in a predictive model developed using a German study population [16], which included age, sex, hypertension-treated, heart failure-treated, valvular heart disease, chronic kidney disease, stroke, hemiplegia, other pulmonary heart diseases, paroxysmal tachycardia, other cardiac arrhythmias, ulcer of lower limb, and personal history of medical treatment (**S2 Table in S1 File**). The model we used was based on a recent study (**S3 Table in S1 File**) [16]. In the model development study, risk factors for incident AF were identified via gradient-boosting with component-wise linear models. From the final gradient-boosted model, an initial list of 43 risk factors for incident AF were identified. Logistic regression models of increasing complexity were then fitted using subsets of the 43 risk factors identified by the gradient-boosted model. An optimal classification model containing 13 of the 43 selected risk factors was identified. These 13 risk factors were defined by three-digit ICD-10 GM10GM (Germany-specific) codes (diagnostic recording) and four- or five-digit ATC (Anatomical Therapeutic Chemical) codes (treatment recording) (**S4 Table in S1 File**).

In this study, risk factors were identified using the same ICD-10, ATC codes, and Read codes (United Kingdom; **S4 and S5 Tables in S1 File**).

Risk factors were identified during the baseline period or at the index date (single code per covariate). This was the same approach as in the model development study. For hypertension-treated, patients were required to have a recording of both an ICD-10 and ATC related code during the baseline period for the risk factor to be recorded as ‘present’ for that patient. This also applied to the heart failure-treated variable.

### Statistical analysis

The analysis was implemented in three phases: 1) model-relatedness investigation i.e. a comparison of risk factors across databases; 2) predictive model specification and external validation; and 3) sensitivity analyses. To explore and compare similarities in variables across five country-level databases, descriptive statistics characterised the presence of the risk factors in both cases and controls. Two-way linear dependence on all risk factors was identified using Pearson’s correlation coefficient. Logistic regression model was then fitted to a binary response (yes/no) for AF diagnosis using the pre-determined risk factors. This predictive model was implemented in all databases.

In the external validation phase, performance metrics were evaluated, including model discrimination. Missing data were treated as absent, which is a common statistical approach to analysing observational data from electronic medical records [17]. Model predictions were added into one of the following categories:

- True positives (TP): Incident AF patients correctly identified by the predictive model as having had the outcome
- False positives (FP): Patients without incident AF incorrectly identified by the predictive model as having had the outcome
- True negatives (TN): Patients without incident AF correctly identified by the predictive model as NOT having had the outcome
- False negatives (FN): Incident AF patients incorrectly identified by the predictive model as NOT having had the outcome

Based on the definitions, the measures of model performance for each validation data source included:

- *True positive rate (Sensitivity/Recall)*: This is ability of the predictive model to correctly identify patients with incident AF

$$True\;positive\;rate=\frac{TP}{TP+FN}$$

- *True negative rate (Specificity)*: This is the ability of the predictive model to correctly identify patients without incident AF

$$True\;negative\;rate=\frac{TN}{TN+FP}$$

- *Positive predictive value (PPV*, *Precision)*: This is the proportion of true positive model predictions out of all positive model predictions

$$PPV=\frac{TP}{TP+FP}$$

The discriminative ability of the model was assessed using receiver operating characteristic (ROC) curves. The area under the curve (AUC) was used to determine the predictive performance of the model. Performance metrics such as PPV are dependent on class imbalance and, therefore, influenced by the AF prevalence in the datasets. To determine the level of clinical burden, PPV, the proportion of true positive model predictions out of all positive model predictions, and number needed to screen, the number of patients that it is necessary to further evaluate to detect one patient with AF were calculated.

In the analysis, the intercepts of all validation models were estimated to approximate the baseline AF risk in the model development population (**S1 File**). In the sensitivity analysis, the intercept was calibrated to each validation dataset.

The performance of the algorithm was also assessed by calculating the Brier score in each data source. This score considers both discrimination and calibration and is a useful summary measure of the overall accuracy of the algorithm. The Brier score can take values between 0 and 1, where a Brier score of 0 would indicate a perfectly predicting model (i.e. 0 error in model prediction).

All analyses were conducted with R version 3.6.1.

## Results

### Patient characteristics

The characteristics of patients with AF who were eligible for the study are highlighted in **Table 1**. Most cases were from Germany (n = 63,562), followed by the UK (n = 42,652), France (n = 7,213), Australia (n = 2,753), and Belgium (n = 1,371), and the majority of controls included in the study were from Germany (n = 1,543,436); the baseline characteristics of both cases and controls are shown in **Table 2**. The distribution of age and sex between AF and controls were comparable in the validation datasets and the original model development study (**S2 Table in S1 File**), although treated hypertension was more prevalent in cases than controls in all databases. The overall prevalence of risk factors in patients with AF differed between country-level databases, although the key risk factors were more common in AF versus control groups. No strong correlation (> ±0.5) was observed between risk factors. The correlation plots are included in the **S2 Fig in S1 File**. The highest proportion of patients with AF was observed in the Australia data set (n = 2,753/51,761, 5.3%), followed by Germany (n = 63,562/1,606,998, 4.0%), the UK (n = 42,652/1,278,423, 3.3%), France (n = 7,213/434,323, 1.7%), and Belgium (n = 1,371/123,464, 1.1%). In comparison, the original German model identified 66,697cases (5.6%) of incident AF.

**Table 1:** Attrition in all databases among A) cases and B) controls.

	United Kingdom	France	Germany	Belgium	Australia
	N (%)	N (%)	N (%)	N (%)	N (%)
**A)**					
**Selection criteria**					
All patients with AF diagnosed from 1/1/2010 to 12/31/2016	135,442 (100.0)	43,305 (100.0)	311,800 (100.0)	13,794 (100.0)	10,374 (100.0)
Patients with index date between 1/1/2013 and 12/31/2016	54,709 (40.4)	11,772 (27.2)	170,100 (54.6)	3,419 (24.8)	4,836 (46.6)
Patients with continuous enrollment during the baseline period (1,095 days before pre-index date)	43,523 (32.1)	7,405 (17.1)	64,735 (20.8)	1,441 (10.4)	2,939 (28.3)
Patients ≥ 45years at index date	42,652 (31.5)	7,213 (16.7)	63,562 (20.4)	1,371 (9.9)	2,753 (26.5)
**Eligible patients**					
Overall	42,652 (100.0)	7,213 (100.0)	63,562 (100.0)	1,371 (100.0)	2,753 (100.0)
By year of index date					
2013	11,893 (27.9)	1,733 (24.0)	18,700 (29.4)	318 (23.2)	767 (27.9)
2014	11,298 (26.5)	1,803 (25.0)	15,893 (25.0)	369 (26.9)	739 (26.8)
2015	10,381 (24.3)	1,833 (25.4)	17,296 (27.2)	347 (25.3)	670 (24.3)
2016	9,080 (21.3)	1,844 (25.6)	11,673 (18.4)	337 (24.6)	577 (21.0)
**B)**					
**Selection criteria**					
All patients without AF diagnosed from 1/1/2010 to 12/31/2016	15,702,404 (100.0)	4,491,194 (100.0)	2,419,168 (100.0)	852,511 (100.0)	2,108,811 (100.0)
Patients with continuous enrolment from January 2010 to earliest of end of study point or to death	3,066,561 (19.5)	822,742 (18.3)	2,300,497 (95.1)	224,713 (26.4)	89,136 (4.2)
Patients with index date between 1/1/2013 and 12/31/2016	3,066,561 (19.5)	822,742 (18.3)	2,300,497 (95.1)	224,713 (26.4)	89,136 (4.2)
Patients with date of death recorded after index date	2,337,506 (14.9)	822,742 (18.3)	2,300,497 (95.1)	224,713 (26.4)	89,136 (4.2)
Patients ≥ 45 years of age at index date	1,235,771 (7.9)	427,110 (9.5)	1,543,436 (63.8)	122,093 (14.3)	49,008 (2.3)
**Eligible patients**					
Overall	1,235,771 (100.0)	427,110 (100.0)	1,543,436 (100.0)	122,093 (100.0)	49,008 (100.0)
By year of index date					
2013	302,581 (24.5)	101,206 (23.7)	371,623 (24.1)	28,971 (23.7)	11,677 (23.8)
2014	305,295 (24.7)	104,818 (24.5)	381,630 (24.7)	30,176 (24.7)	11,955 (24.4)
2015	310,470 (25.1)	108,764 (25.5)	390,719 (25.3)	31,025 (25.4)	12,472 (25.4)
2016	317,425 (25.7)	112,322 (26.3)	399,464 (25.9)	31,921 (26.1)	12,904 (26.3)

Provided are numbers (%). Abbreviation: AF = atrial fibrillation

**Table 2:** Model relatedness between the original study and each of the validation databases.

Covariate	Model development population N = 1,182,182		United Kingdom N = 1,278,423		France N = 434,323		Germany N = 1,606,998		Belgium N = 123,464		Australia N = 51,761	
	No AF	AF	No AF	AF	No AF	AF	No AF	AF	No AF	AF	No AF	AF
Cohort Size, N (%)*	1,115,485 (94.36)	66,697 (5.64)	1,235,771 (96.66)	42,652 (3.34)	427,107 (98.34)	7,213 (1.66)	1,543,436 (96.04)	63,562 (3.96)	122,093 (98.89)	1,371 (1.11)	49,008 (94.68)	2,753 (5.32)
Age (years), mean (SD)	61.1 (11.5)	74.7 (11.5)	61.78 (11.72)	74.70 (10.76)	61.84 (11.58)	74.88 (10.63)	63.56 (11.61)	74.92 (9.91)	62.07 (11.67)	72.94 (11.78)	62.01 (11.44)	72.93 (11.40)
Age (years), categorical n (%)												
45–49	(64.6)	(18)	206,770 (16.73)	716 (1.68)	68,748 (16.10)	95 (1.32)	201,901 (13.08)	966 (1.52)	18,711 (15.33)	48 (3.50)	7320 (14.94)	77 (2.80)
50–54			207,714 (16.81)	1,391 (3.26)	69,941 (16.38)	234 (3.24)	225,921 (14.64)	1,729 (2.72)	20,178 (16.53)	61 (4.45)	7,833 (15.98)	123 (4.47)
55–59			182,499 (14.77)	2,034 (4.77)	65,165 (15.26)	348 (4.82)	213,196 (13.81)	2,719 (4.28)	18,946 (15.52)	93 (6.78)	8,012 (16.35)	181 (6.57)
60–64			161,193 (13.04)	3,262 (7.65)	59,545 (13.94)	580 (8.04)	202,724 (13.13)	4,489 (7.06)	17,105 (14.01)	144 (10.50)	7,045 (14.38)	271 (9.84)
65–69	(19.8)	(25.7)	158,870 (12.86)	5,339 (12.52)	55,861 (13.08)	912 (12.64)	177,876 (11.52)	6,061 (9.54)	15,281 (12.52)	170 (12.40)	6,177 (12.60)	349 (12.68)
70–74			117,909 (9.54)	6,639 (15.57)	37,765 (8.84)	1,022 (14.17)	189,484 (12.28)	10,686 (16.81)	10,759 (8.81)	183 (13.35)	4,675 (9.54)	434 (15.76)
75–79	(15.6)	(56.4)	89,770 (7.26)	7,787 (18.26)	30,932 (7.24)	1,325 (18.37)	185,926 (12.05)	15,059 (23.69)	9,268 (7.59)	220 (16.05)	3,655 (7.46)	450 (16.35)
80–84			60,806 (4.92)	7,442 (17.45)	22,689 (5.31)	1,299 (18.01)	93,733 (6.07)	11,582 (18.22)	6,766 (5.54)	204 (14.88)	2,384 (4.86)	396 (14.38)
85–89			33,223 (2.69)	5,149 (12.07)	11,734 (2.75)	951 (13.18)	40,606 (2.63)	7,415 (11.67)	3,593 (2.94)	161 (11.74)	1,364 (2.78)	312 (11.33)
90+			17,017 (1.38)	2,893 (6.78)	4,727 (1.11)	447 (6.20)	12,069 (0.78)	2,856 (4.49)	1,486 (1.22)	87 (6.35)	543 (1.11)	160 (5.81)
Sex, n (%)												
Female	(51.9)	(46.0)	647,954 (52.43)	19,585 (45.92)	233,322 (54.63)	3,280 (45.47)	908,454 (58.86)	30,487 (47.96)	66,460 (54.43)	649 (47.34)	26,697 (54.47)	1,272 (46.20)
Male	-	-	587,817 (47.57)	23,067 (54.08)	193,785 (45.37)	3,933 (54.53)	634,982 (41.14)	33,075 (52.04)	55,633 (45.57)	722 (52.66)	22,311 (45.53)	1,481 (53.80)
Hypertension treated, n (%)												
Yes	(49.3)	(81.8)	109,319 (8.85)	10,142 (23.78)	30,799 (7.21)	2,013 (27.91)	122,023 (7.91)	25,366 (39.91)	8,543 (7.00)	250 (18.23)	6,706 (13.68)	992 (36.03)
No	-	-	1,126,452 (91.15)	32,510 (76.22)	396,308 (92.79)	5,200 (72.09)	1,421,413 (92.09)	38,196 (60.09)	113,550 (93.00)	1,121 (81.77)	42,302 (86.32)	1,761 (63.97)
Heart failure treated, n (%)												
Yes	(4.7)^a^	(21.3)	8,553 (0.69)	3,299 (7.73)	1,927 (0.45)	444 (6.16)	23,373 (1.51)	11,578 (18.22)	1,273 (1.04)	140 (10.21)	536 (1.09)	350 (12.71)
No	-	-	1,227,218 (99.31)	39,353 (92.27)	425,180 (99.55)	6,769 (93.84)	1,520,063 (98.49)	51,984 (81.78)	120,820 (98.96)	1,231 (89.79)	48,472 (98.91)	2,403 (87.29)
Valvular heart disease, n (%)												
Yes	(5.8)	(18.6)	6,929 (0.56)	2,074 (4.86)	8,249 (1.93)	537 (7.44)	20,415 (1.32)	8,366 (13.16)	785 (0.64)	41 (2.99)	164 (0.33)	52 (1.89)
No	-	-	1,228,842 (99.44)	40,578 (95.14)	418,858 (98.07)	6,676 (92.56)	1,523,021 (98.68)	55,196 (86.84)	121,308 (99.36)	1,330 (97.01)	48,844 (99.67)	2,701 (98.11)
Chronic kidney disease, n (%)												
Yes	(8.1)	(16.7)	36,141 (2.92)	3,536 (8.29)	2,016 (0.47)	126 (1.75)	17,141 (1.11)	5,044 (7.94)	780 (0.64)	54 (3.94)	453 (0.92)	86 (3.12)
No	-	-	1,199,630 (97.08)	39,116 (91.71)	425,091 (99.53)	7,087 (98.25)	1,526,295 (98.89)	58,518 (92.06)	121,313 (99.36)	1,317 (96.06)	48,555 (99.08)	2,667 (96.88)
Stroke, not specified as hemorrhage or infarction, n (%)												
Yes	(0.5)	(4.0)	11,722 (0.95)	1,600 (3.75)	3,997 (0.94)	302 (4.19)	8,302 (0.54)	2,467 (3.88)	1,249 (1.02)	51 (3.72)	591 (1.21)	124 (4.50)
No	-	-	1,224,049 (99.05)	41,052 (96.25)	423,110 (99.06)	6,911 (95.81)	1,535,134 (99.46)	61,095 (96.12)	120,844 (98.98)	1,320 (96.28)	48,417 (98.79)	2,629 (95.50)
Hemiplegia, n (%)												
Yes	-	-	117 (0.01)	24 (0.06)	622 (0.15)	25 (0.35)	4,143 (0.27)	1,140 (1.79)	67 (0.05)	4 (0.29)	12 (0.02)	4 (0.15)
No	-	-	1,235,654 (99.99)	42,628 (99.94)	426,485 (99.85)	7,188 (99.65)	1,539,293 (99.73)	62,422 (98.21)	122,026 (99.95)	1,367 (99.71)	48,996 (99.98)	2,749 (99.85)
Other pulmonary heart diseases, n (%)												
Yes	-	-	445 (0.04)	143 (0.34)	903 (0.21)	63 (0.87)	1,795 (0.12)	1,388 (2.18)	80 (0.07)	7 (0.51)	16 (0.03)	18 (0.65)
No	-	-	1,235,326 (99.96)	42,509 (99.66)	426,204 (99.79)	7150 (99.13)	1,541,641 (99.88)	62,174 (97.82)	122,013 (99.93)	1,364 (99.49)	48,992 (99.97)	2,735 (99.35)
Other cardiac arrhythmias, n (%)												
Yes	(7.1)	(23.2)	5,887 (0.48)	1,680 (3.94)	10,189 (2.39)	1,551 (20.95)	29,564 (1.92)	9,437 (14.85)	3,214 (2.63)	208 (15.17)	37 (0.08)	401 (14.57)
No			1,229,884 (99.52)	40,972 (96.06)	416,918 (97.61)	5,702 (79.05)	1,513,872 (98.08)	54,125 (85.15)	118,879 (97.37)	1,163 (84.83)	48,971 (99.92)	2,352 (85.43)
Paroxysmal tachycardia, n (%)												
Yes	-	-	1,856 (0.15)	684 (1.60)	3,204 (0.75)	225 (3.12)	5,659 (0.37)	1,777 (2.80)	1,403 (1.15)	200 (14.59)	150 (0.31)	63 (2.29)
No	-	-	1,233,915 (99.85)	41,968 (98.4)	423,903 (99.25)	6,988 (96.88)	1,537,777 (99.63)	61,785 (97.20)	120,690 (98.85)	1,171 (85.41)	48,858 (99.69)	2,690 (97.71)
Ulcer of lower limb, not elsewhere classified, n (%)												
Yes	-	-	1,086 (0.09)	131 (0.31)	1160 (0.27)	105 (1.46)	6,284 (0.41)	1647 (2.59)	0 (0)	0 (0)	1,004 (2.05)	89 (3.23)
No	-	-	1,234,685 (99.91)	42,521 (99.69)	425,947 (99.73)	7,108 (98.54)	1,537,152 (99.59)	61,915 (97.41)	122,093 (100.00)	1,371 (100.00)	48,004 (97.95)	2,664 (96.77)
Personal history of medical treatment, n (%)												
Yes	-	-	51,693 (4.18)	2,410 (5.65)	2,448 (0.57)	234 (3.24)	16,501 (1.07)	11,886 (18.70)	0 (0)	0 (0)	1,056 (2.15)	237 (8.61)
No	-	-	1,184,078 (95.82)	40,242 (94.35)	424,659 (99.43)	6,979 (96.76)	1,526,935 (98.93)	51,676 (81.30)	122,093 (100.00)	1,371 (100.00)	47,952 (97.85)	2,516 (91.39)

*Percentages for Cohort Size calculated for the whole study population (AF + no AF). All other percentages are calculated for their respective columns.

^a^The values in parentheses for Model development are percentages

Abbreviations: AF, atrial fibrillation; SD, standard deviation.

### Model relatedness between original model and validation databases

Patients with AF were more likely to have hypertension, heart failure or other cardiac conditions than controls in all validation datasets than the model development data. Certain comorbidities of interest were not significantly associated with AF in the validation datasets, such as chronic kidney disease in the France dataset, hemiplegia and other pulmonary heart disease in the Belgium dataset, and chronic kidney disease and lower limb ulcers in the Australian dataset.

### Model performance

**Fig 2:**
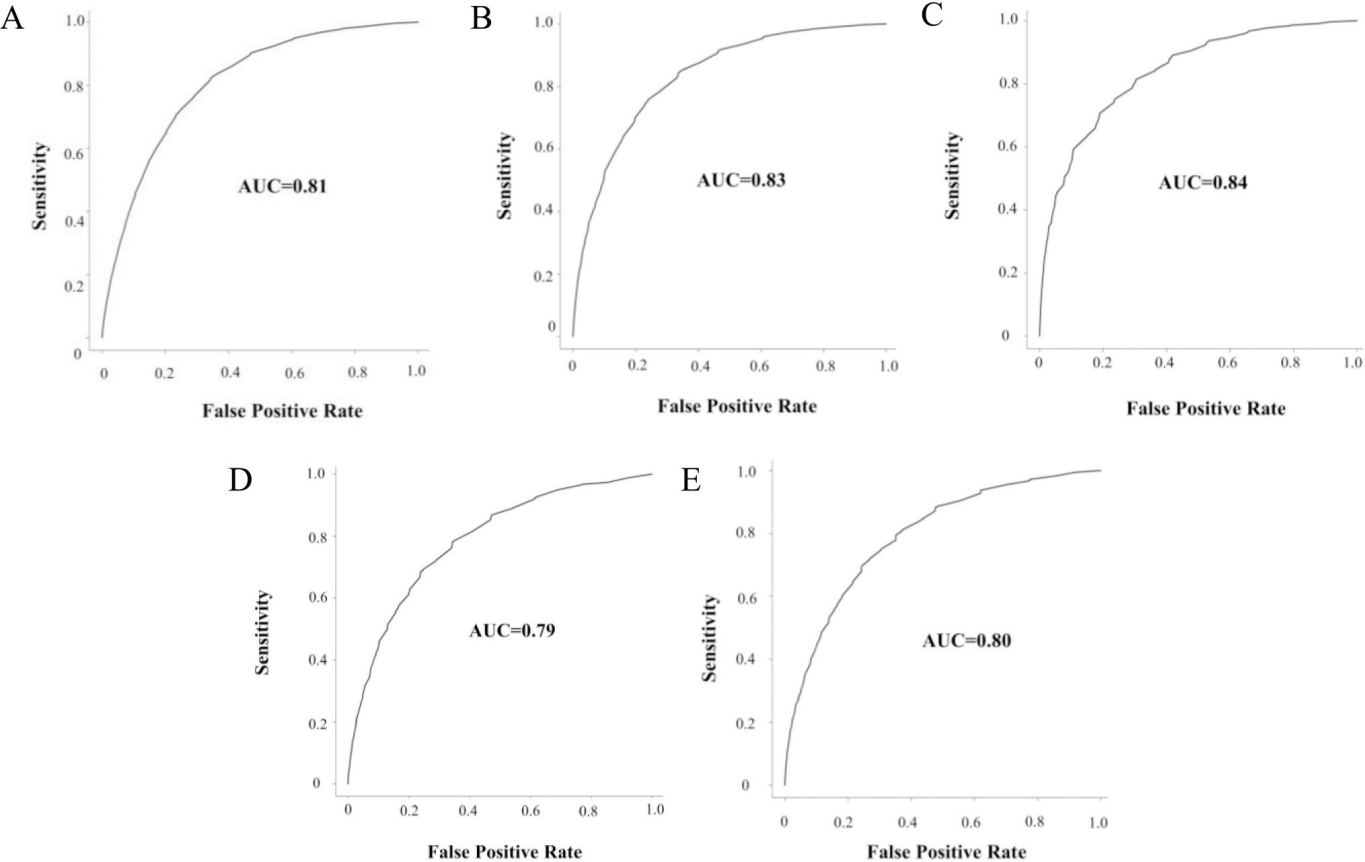
Receiver operating characteristic (ROC) curve for IQVIA Medical Research Data (IMRD) in A) UK B) France C) Germany D) Belgium E) Australia.

The original model had a sensitivity of 80% and a specificity of 72%. In the validation datasets at approximately 80% sensitivity, the model reported similar specificity within an acceptable range (France: 67% and UK: 71%), although the specificity in the Belgium dataset was 59% (**Table 3**). The original model with 80% sensitivity had a PPV of 14% and required 7 patients to be screened; in the validation datasets, PPV ranged from 2% (Belgium) to 16% (Germany), which required 50 and 6 patients to be screened, respectively. This was related to the prevalence of AF in the databases. The AUC in the validation datasets ranged from 0.79 (Belgium) to 0.84 (Germany), comparable to the original model, which had an AUC of 0.83 (**Fig 2**). All ROC curves bend to the top-left corner indicating good model performance. Brier scores are reported in **S6 Table in S1 File**.

**Table 3:** Measures of predictive performance of the model at ~80% sensitivity.

	United Kingdom	France	Germany	Belgium	Australia	Germany Sensitivity	France Sensitivity
**80% Sensitivity**							
True positives	32,667	5,978	50,960	1,118	2,192	44,541	6,045
False positives	360,555	141,818	460,659	49,798	17,254	236,824	128,443
True negatives	875,216	285,289	1,082,777	72,295	31,754	875,265	242,391
False negatives	9,985	1,235	12,602	253	561	11,210	1,244
True positive rate	0.77	0.83	0.80	0.82	0.80	0.80	0.83
True negative rate	0.71	0.67	0.70	0.59	0.65	0.79	0.65
Positive predictive value	0.08	0.04	0.10	0.02	0.11	0.16	0.04

### Sensitivity results

The shift in study time period did not significantly change the baseline characteristics observed in patients with AF versus controls in the Germany and France datasets. However, patients in the German dataset had increased odds for having a personal history of medication for AF (OR: 9.45, 95% CI: 9.17−9.73) and decreased odds in other cardiac arrhythmias (OR: 3.77, 95%: 3.65−3.88). In the France data set, patients had increased odds for having paroxysmal tachycardia (OR: 2.50, 95% CI: 2.15−2.91). At 80% sensitivity, the PPV increased slightly in the Germany dataset, but remained the same for the France dataset. Compared to the main study, the ROC curves in the sensitivity analysis a similar AUC in the Germany and France dataset (**S3A and S3B Fig in S1 File**). Overall, the shift in study time period did not markedly alter the model validation results for both datasets.

## Discussion

This study sought to assess the predictive capability of one model to screen for AF in several large databases. The validation datasets demonstrated a comparable performance in terms of sensitivity and specificity and AUC to the original German model. When controlling for AF, prevalence PPV was also similar. Predicting events such as AF with low prevalence is challenging and classic model performance metrics alone are not appropriate to evaluate the value of screening tools in this setting. Assessing algorithms in low prevalence diseases should consider benefit and burden to the system. This can be evaluated using the PPV and number needed to screen to assess the potential burden of clinical implementation as a result of false-positives, and metrics, such as sensitivity to assess the benefit of the algorithm in identifying positive cases [18]. In this analysis, the prevalence of AF varied widely between these datasets, and low prevalence affected some metrics, such as PPV, although sensitivity, specificity, and ROC were not affected by prevalence. Therefore, the model performs variably if the prevalence of AF is relatively low, however, performance may also be related to how variables were recorded in the datasets, in addition to differences in patient populations across datasets.

The higher sensitivity of the model can offer increased clinical value in screening for AF. This model can predict AF using electronic medical records in a primary care setting as demonstrated by the validation datasets in this study, and claims data including in hospital settings as shown by the original model development study, which supports implementation efforts in different care settings that utilise ICD-10 coding.

Algorithms that predict AF using clinical factors have shown to be relatively accurate in several studies. For instance, a 10-year risk score developed to identify AF in Caucasians and African Americans showed moderate discrimination (AUC = 0.78) [19]. Another study using machine learning to predict AF demonstrated 75% sensitivity compared to the CHARGE-AF model, thereby reducing the number of patients needed to be screened [14]. Further, a predictive model derived from pooled individual-level primary care data from several large US population cohorts was used to predict AF [20], and showed good discrimination in two validation cohorts [21]. Therefore, studies have increasingly implemented predictive algorithms using electronic health records for identifying AF [22, 23] and stratifying the risk for stroke [24]. Using routine clinical data to screen for AF enables researchers and clinicians to make earlier diagnoses of AF, assess the clinical burden over time and to improve the timeliness of guideline-recommended treatment. Algorithms can be integrated in clinical decision support systems to analyze data within electronic health care records automatically and to provide prompts and reminders to assist health care providers. This strategy may augment the implementation of guideline recommendations in clinical practice and may increase cost-effectiveness of pre-selecting patients at-risk for AF by using algorithms [25]. In the current study, the model was able to identify approximately 80% of all positive cases and 71% of negative cases correctly. Four of the five calibrated models performed at expected real-world prevalence rates but deviated when prevalence decreased below proportions one would expect to see in a real-world setting. It is likely that the model could be implemented in different settings and with different designs in a real-world setting. For example, Schnabel et al. (2022) also validated the model in a prospective cohort in ambulatory patients with cardiovascular risk [16]. If implemented in electronic health records, patients at high risk for AF could be identified and invited for confirmatory screening similar to a clinical study designed by Hill et al. [26]. The most suitable approach for implementation will likely depend on the healthcare system, provider, and data infrastructure.

Some limitations should be acknowledged. Controls required continuous enrolment from the start of the study period to the end of the study period or death. This was to ensure that controls did not develop AF during the entire study period. Continuous enrolment may cause generalizability issues, particularly in claims databases from countries such as the US where patients are more likely to move between health plans. However, continuous enrolment is likely to be limited in countries with EMR and national healthcare systems such as countries in Europe. Additionally, bias may be introduced into database studies because of miscoding. In this study, it is feasible that AF might have been coded as “other cardiac arrhythmias". However, AF is a well-defined and common arrhythmia which is known to physicians across specialties. Coding of AF is important for re-imbursement. Misclassification can certainly occur but is less likely for this arrhythmia. Moreover, data presented in this paper utilize Western countries only, and, therefore, may not be generalisable to other countries. Given that this study had a retrospective study design, future prospective studies would help to assess the utility of these models through real-world implementation.

## Conclusions

Given the serious health consequences of AF, screening algorithms offer targeted ways to identify at-risk patients using electronic health records and may help to improve the cost-effectiveness of screening programs. Based on the results of this study, the algorithm can be transferred to databases from different healthcare systems.

## Supporting information

S1 File (PDF)
