# Supplementary material for: Performance of an electronic health record-based predictive model to identify patients with atrial fibrillation across countries
Source: PLoS One. 2022 Jul 8;17(7):e0269867. doi: 10.1371/journal.pone.0269867 (PMC9269467; doi:10.1371/journal.pone.0269867)
Supplement: S1 File — (PDF) [file pone.0269867.s001.pdf]

SUPPLEMENTAL MATERIAL

S1 File

The following method was used to approximate the intercept of the original model:

The β_1, β_2, $\beta_{3,\ldots,}$ β_{13} coefficients for each of the model covariates can be determined using logistic regression formula below.

$$Log(p/(1-p)) = 0 + \beta_1 X_1 + \beta_2 X_2 + \beta_3 X_3 + \ldots + \beta_{13} X_{13}$$

The range was defined using α from -5.0 to +5.0 in increments of 0.1. The equation used to estimate the baseline *event rate* $p = Exp(-5.0 + \beta_1 X_1 + \beta_2 X_2 + \beta_3 X_3 + \ldots + \beta_{13} X_{13}) / (1+Exp(-5.0+ \beta_1 X_1 + \beta_2 X_2 + \beta_3 X_3 + \ldots + \beta_{13} X_{13}))$. Compared to the original German study, the event rate was 5.979% = 66,697/1,115,485.

If the rates were different, the α was changed to -4.9 and this was repeated until close enough rates were obtained. Depending on the baseline risk for AF, the rates were revised in each database. This intercept estimation method was applied to the German validation database, and the intercept value obtained was fixed for all other databases.

In the sensitivity analysis, the intercept was estimated, or re-calibrated, in each database by fitting the logistic regression model with all coefficients that were fixed from the original study to the baseline AF risk for each respective validation database. The original predictive model, as a R/Python object, was not available, and therefore, we had to consider how to model the intercept for the validation databases.

Supplemental Tables

S1 Table. Comparison of datasets used in the study

Country	Data set	Type	Details
**Germany**	InGef (dataset for model development)	Claims (approximately 60 German statutory health insurance providers (SHIs))	Representative data set of 4,350,891 was extracted. In-and-out-patient treatment, prescribed and dispensed medications, sick leave and benefits, prescribed and dispensed medical devices and therapies, as well as demographic information such as age, sex, and location.
**Belgium**	LPD	Electronic medical record database (primary care)	1.1M patient records and 17.6M prescription orders. Data spanning from 2005 onwards.
**France**	LPD	Electronic medical record database (primary care)	7.8M patient records and 236M prescription orders written from GPs, 4M patient records and 23.5M prescription orders written from specialists.
**Germany**	Disease Analyser	Electronic medical record database (primary care)	~34M EMRs, >300M prescription orders. Data spans from 1992 onwards
**UK**	IQVIA Medical Research Data (previously known as THIN)	Electronic medical record database (primary care)	16M patient records, 3M active patients, approximately 6% of the UK population. Data spanning from 1994.

**Australia**	LPD	Electronic medical record database (primary care)	~1.4M patient records with at least one visit. LPD is at a national level. Data spans from 2006.

Abbreviations: InGef, Institute for Applied Healthcare Research Berlin; LPD, longitudinal patient data; THIN, The Health Improvement Network

S2 Table. Model relatedness between the original study and each of the validation databases

Risk factors	Model development population	United Kingdom	France	Germany	Belgium	Australia	Germany (Time sensitivity)	France (Time sensitivity)
	Odds Ratio (95% CI)	Odds Ratio (95% CI)	Odds Ratio (95% CI)	Odds Ratio (95% CI)	Odds Ratio (95% CI)	Odds Ratio (95% CI)	Odds Ratio (95% CI)	Odds Ratio (95% CI)
Intercept	*Unavailable*	0.05 (0.05 - 0.05)	0.02 (0.02-0.02)	0.03 (0.03 - 0.03)	0.01 (0.01 - 0.01)	0.06 (0.05 - 0.07)	**0.02 (0.02 - 0.03)**	**0.02 (0.02 - 0.03)**
**Age**								
Age 75-79	Ref	Ref	Ref	Ref	Ref	Ref	Ref	Ref
Age 45-49	0.08 (0.08 - 0.09)*	0.05 (0.05 - 0.05)*	0.05 (0.04 - 0.06)*	0.11 (0.10 - 0.11)*	0.15 (0.11 - 0.2)*	0.10 (0.08 - 0.13)*	0.09 (0.08 - 0.10)*	0.06 (0.05 - 0.07)*
Age 50-54	0.13 (0.12 - 0.13)*	0.09 (0.09 - 0.10)*	0.11 (0.10 - 0.13)*	0.16 (0.15 - 0.16)*	0.17 (0.13 - 0.22)*	0.16 (0.12 - 0.19)*	0.13 (0.12 - 0.14)*	0.10 (0.09 - 0.12)*
Age 55-59	0.19 (0.18 - 0.2)*	0.15 (0.15 - 0.16)*	0.17 (0.15 - 0.19)*	0.23 (0.22 - 0.24)*	0.26 (0.2 - 0.33)*	0.21 (0.18 - 0.26)*	0.20 (0.19 - 0.21)*	0.19 (0.17 - 0.21)*
Age 60-64	0.3 (0.29 - 0.31)*	0.27 (0.26 - 0.28)*	0.29 (0.26 - 0.32)*	0.37 (0.35 - 0.38)*	0.43 (0.35 - 0.54)*	0.33 (0.28 - 0.39)*	0.30 (0.29 - 0.32)*	0.28 (0.25 - 0.31)*
Age 65-69	0.46 (0.45 - 0.48)*	0.43 (0.41 - 0.44)*	0.45 (0.41 - 0.49)*	0.52 (0.51 - 0.54)*	0.54 (0.44 - 0.67)*	0.47 (0.40 - 0.56)*	0.48 (0.46 - 0.50)*	0.45 (0.42 - 0.50)*
Age 70-74	0.71 (0.69 - 0.73)*	0.68 (0.66 - 0.71)*	0.7 (0.64 - 0.76)*	0.79 (0.77 - 0.81)*	0.78 (0.64 - 0.96)	0.78 (0.68 - 0.91)	0.72 (0.70 - 0.75)*	0.69 (0.64 - 0.75)*
Age 80-84	1.45 (1.41 - 1.49)*	1.37 (1.32 - 1.42)*	1.21 (1.12 - 1.32)*	1.32 (1.28 - 1.36)*	1.25 (1.03 - 1.53)	1.24 (1.06 - 1.46)	1.37 (1.33 - 1.41)*	1.18 (1.08 - 1.28)
Age 85-89	1.91 (1.85 - 1.97)*	1.70 (1.64 - 1.77)*	1.6 (1.46 - 1.75)*	1.73 (1.67 - 1.79)*	1.87 (1.51 - 2.32)*	1.55 (1.31 - 1.85)*	1.93 (1.86 - 2.00)*	1.63 (1.49 - 1.78)*
Age 90+	2.24 (2.15 - 2.34)*	1.99 (1.90 - 2.08)*	1.94 (1.72 - 2.18)*	2.13 (2.02 - 2.24)*	2.41 (1.84 - 3.15)*	2.05 (1.64 - 2.56)*	2.73 (2.60 - 2.86)*	2.03 (1.81 - 2.27)*
**Sex**								
Female	Reference	Reference	Reference	Reference	Reference	Reference	Reference	Reference
Male	1.52 (1.49 - 1.54)*	1.51 (1.48 - 1.55)*	1.65 (1.57 - 1.74)*	1.51 (1.49 - 1.54)*	1.54 (1.38 - 1.72)*	1.44 (1.32 - 1.57)*	1.67 (1.64 - 1.71)*	1.62 (1.54 - 1.70)*
**Hypertension** treated								
No	Reference	Reference	Reference	Reference	Reference	Reference	Reference	Reference
Yes	1.76 (1.72 - 1.79)*	1.62 (1.58 - 1.66)*	2.10 (1.98 - 2.23)*	2.82 (2.76 - 2.88)*	1.26 (1.07 - 1.47)*	1.83 (1.66 - 2.01)*	3.04 (2.97 - 3.11)*	2.03 (1.91 - 2.15)*
**Heart failure** treated								
No	Reference	Reference	Reference	Reference	Reference	Reference	Reference	Reference

Yes	1.54 (1.5 - 1.58)*	4.25 (4.06 - 4.45)*	3.16 (2.80 - 3.57)*	2.63 (2.55 - 2.71)*	3.09 (2.49 - 3.83)*	4.93 (4.20 - 5.79)*	3.48 (3.38 - 3.58)*	3.51 (3.11 - 3.96)*
**Valvular heart** disease								
No	Reference	Reference	Reference	Reference	Reference	Reference	Reference	Reference
Yes	1.42 (1.39 - 1.46)*	3.09 (2.92 - 3.27)*	1.75 (1.58 - 1.93)*	3.32 (3.21 - 3.43)*	1.78 (1.25 - 2.54)*	2.98 (2.04 - 4.35)*	3.38 (3.26 - 3.49)*	1.68 (1.52 - 1.86)*
**Chronic kidney** disease								
No	Reference	Reference	Reference	Reference	Reference	Reference	Reference	Reference
Yes	1.21 (1.18 - 1.24)*	1.13 (1.08 - 1.17)*	0.97 (0.79 - 1.18)	1.60 (1.53 - 1.66)*	1.67 (1.21 - 2.32)*	1.08 (0.82 - 1.42)	1.37 (1.32 - 1.42)*	1.07 (0.90 - 1.29)
**Stroke, not specified as hemorrhage or infarction**								
No	Reference	Reference	Reference	Reference	Reference	Reference	Reference	Reference
Yes	2.43 (2.29 - 2.57)*	1.75 (1.65 - 1.85)*	1.96 (1.72 - 2.23)*	2.12 (2.00 - 2.25)*	1.83 (1.35 - 2.48)*	1.93 (1.54 - 2.43)*	1.71 (1.60 - 1.83)*	1.87 (1.65 - 2.12)*
**Hemiplegia**								
No	Reference	Reference	Reference	Reference	Reference	Reference	Reference	Reference
Yes	3.04 (2.86 - 3.23)*	3.27 (2.02 - 5.30)*	1.21 (0.79 - 1.85)	1.94 (1.78 - 2.11)*	1.32 (0.41 - 4.23)*	3.50 (1.02 - 11.99)*	1.50 (1.38 - 1.64)*	1.18 (0.77 - 1.82)
**Other pulmonary heart diseases**								
No	Reference	Reference	Reference	Reference	Reference	Reference	Reference	Reference
Yes	1.6 (1.51 - 1.69)*	1.89 (1.52 - 2.34)*	2.04 (1.54 - 2.70)*	2.89 (2.64 - 3.17)*	1.87 (0.78 - 4.5)*	7.66 (3.51 - 16.75)*	3.21 (2.93 - 3.51)*	2.22 (1.68 - 2.94)*
**Other cardiac arrhythmias**								
No	Reference	Reference	Reference	Reference	Reference	Reference	Reference	Reference
Yes	2.2 (2.11 - 2.3)*	2.91 (2.73 - 3.09)*	4.95 (4.64 - 5.28)*	3.77 (3.65 - 3.88)*	3.05 (2.59 - 3.6)*	307.30 (214.80 - 439.64)*	2.82 (2.72 - 2.92)*	2.03 (1.73 - 2.39)*
**Paroxysmal tachycardia**								
No	Reference	Reference	Reference	Reference	Reference	Reference	Reference	Reference
Yes	2.11 (2.07 - 2.16)*	7.52 (6.79 - 8.33)*	2.50 (2.15 - 2.91)*	3.81 (3.56 - 4.08)*	8.46 (7.11 - 10.07)*	4.59 (3.16 - 6.67)*	3.37 (3.13 - 3.63)*	4.58 (4.29 - 4.90)*

**Ulcer of lower limb, not elsewhere classified**								
No	Reference	Reference	Reference	Reference	Reference	Reference	Reference	Reference
Yes	1.65 (1.56 - 1.73)*	1.48 (1.22 - 1.80)*	2.07 (1.66 - 2.58)*	1.54 (1.43 - 1.65)*	Not applicable	1.17 (0.91 - 1.51)	1.99 (1.86 - 2.12)*	1.73 (1.35 - 2.22)*
**Personal history of medical treatment**								
No	Reference	Reference	Reference	Reference	Reference	Reference	Reference	Reference
Yes	1.62 (1.58 - 1.65)*	1.24 (1.18 - 1.29)*	2.52 (2.16 - 2.94)*	9.45 (9.17 - 9.73)*	Not applicable	2.00 (1.69 - 2.37)*	14.16 (13.74 - 14.58)*	2.24 (1.91 - 2.63)*

*=p <0.0001

S3 TableTable. AF Prediction Model [a] – Schnabel et al, 2022

Risk Factor	Odds Ratio (95% CI)	p-value
Age 75-79	Reference	
Age 45-49	0.08 (0.08 - 0.09)	<.0001
Age 50-54	0.13 (0.12 - 0.13)	<.0001
Age 55-59	0.19 (0.18 - 0.2)	<.0001
Age 60-64	0.3 (0.29 - 0.31)	<.0001
Age 65-69	0.46 (0.45 - 0.48)	<.0001
Age 70-74	0.71 (0.69 - 0.73)	<.0001
Age 80-84	1.45 (1.41 - 1.49)	<.0001
Age 85-89	1.91 (1.85 - 1.97)	<.0001
Age 90+	2.24 (2.15 - 2.34)	<.0001
Sex [male vs. female]	1.52 (1.49 - 1.54)	<.0001
Hypertension treated [yes vs. no]	1.76 (1.72 - 1.79)	<.0001
Heart failure treated [yes vs. no]	1.54 (1.5 - 1.58)	<.0001
Valvular heart disease [yes vs. no]	1.42 (1.39 - 1.46)	<.0001
Chronic kidney disease [yes vs. no]	1.21 (1.18 - 1.24)	<.0001
Stroke, not specified as hemorrhage or infarction [yes vs. no]	2.43 (2.29 - 2.57)	<.0001
Hemiplegia [yes vs. no]	3.04 (2.86 - 3.23)	<.0001
Other pulmonary heart diseases [yes vs. no]	1.6 (1.51 - 1.69)	<.0001
Paroxysmal tachycardia [yes vs. no]	2.2 (2.11 - 2.3)	<.0001
Other cardiac arrhythmias [yes vs. no]	2.11 (2.07 - 2.16)	<.0001
Ulcer of lower limb, not elsewhere classified [yes vs. no]	1.65 (1.56 - 1.73)	<.0001
Personal history of medical treatment [yes vs. no]	1.62 (1.58 - 1.65)	<.0001

[a] The intercept used was -2.5

S4 Table. ICD-10 and ATC codes.

Variable	Code Ontology[a]
Case definition	
Atrial fibrillation diagnosis	ICD-10: I48.0; I48.1;I48.2;I48.9
Risk factors	
Hypertension treated	ICD-10: I10, I11; I12; I13; 115 ATC: C02; C03
Heart failure treated	ICD-10: I50, ATC: C09A; C07; C03DA
Valvular heart disease	ICD-10: I05.-I08.; I34.-I39.
Chronic renal insufficiency	ICD-10: N18
Stroke (not specified as hameorrhage or infarction)	ICD-10: I64
Hemiplegia	ICD-10: G81
Other pulmonary heart diseases	ICD-10: I27
Paroxysmal tachycardia	ICD-10: I47
Other cardiac arrhythmias	ICD-10: I49
Ulcer of lower limb; not elsewhere classified	ICD-10: L97
Personal history of medical treatment	ICD-10: Z92

Abbreviations: ATC, anatomical therapeutic chemical; ICD-10, international classification of diseases 10th edition

[a]ICD-10 codes based on Schnabel, 2022[16] and Van den Ham, 2021[27]

S5 Table 55. ICD-10 codes and Read codes[a]

Atrial Fibrillation diagnosis:

Code	Description	Code	Description
G573.00	Atrial fibrillation and flutter	793M300	Perc translum ablat conduct sys heart for atrial flutter NEC
G573000	Atrial fibrillation	8CMW200	Atrial fibrillation care pathway
G573100	Atrial flutter	8HTy.00	Referral to atrial fibrillation clinic
G573200	Paroxysmal atrial fibrillation	8OAD.00	Provision of written information about atrial fibrillation
G573300	Non-rheumatic atrial fibrillation	9hF..00	Exception reporting: atrial fibrillation quality indicators
G573400	Permanent atrial fibrillation	9hF1.00	Excepted from atrial fibrillation qual indic: Inform dissent
G573500	Persistent atrial fibrillation	9Os..00	Atrial fibrillation monitoring administration
G573600	Paroxysmal atrial flutter	9Os0.00	Atrial fibrillation monitoring first letter
G573z00	Atrial fibrillation and flutter NOS	9Os1.00	Atrial fibrillation monitoring second letter
662S.00	Atrial fibrillation monitoring	9Os2.00	Atrial fibrillation monitoring third letter
6A9..00	Atrial fibrillation annual review	9Os3.00	Atrial fibrillation monitoring verbal invite
7930000	Open ablation of atrioventricular node	9Os4.00	Atrial fibrillation monitoring telephone invite
7934000	Percutaneous transluminal ablation of atrioventricular node	G573700	Chronic atrial fibrillation
7934500	Percutaneous transluminal ablation of atrial wall	G573800	Typical atrial flutter
7934800	Percutaneous transluminal ablation of atrial wall NEC	G573900	Atypical atrial flutter
7936A00	Implant intravenous pacemaker for atrial fibrillation	3272.00	ECG: atrial fibrillation
793M100	Perc transluminal ablation of atrial wall for atrial flutter	3273.00	ECG: atrial flutter

Valvular heart disease

Code	Description	Code	Description
14S4.00	H/O: heart valve recipient	G121.00	Rheumatic aortic insufficiency
14T3.00	H/O: artificial heart valve	G121.12	Aortic regurgitation - rheumatic
1952.11	Regurgitation	G122.00	Rheumatic aortic stenosis with insufficiency
24D9.00	O/E - tricuspid murmur	G12z.00	Rheumatic aortic valve disease NOS
7902000	Correct Fallot tetralogy- valved right ventr outflow conduit	G13..00	Diseases of mitral and aortic valves
7902011	Rep tetralog Fallot valved right ventricular outflow conduit	G130.00	Mitral and aortic stenosis
7902500	Repair of tetralogy of Fallot with absent pulmonary valve	G131.00	Mitral stenosis and aortic insufficiency
790D.00	Creation of valved cardiac conduit	G131.13	Mitral stenosis and aortic incompetence
790D000	Creation valved conduit between heart atrium and ventricle	G131.14	Mitral stenosis and aortic regurgitation
790D100	Creation of valved conduit between right atrium+pulm artery	G132.00	Mitral insufficiency and aortic stenosis
790D200	Creation of valved conduit between right ventr +pulm artery	G132.12	Mitral incompetence and aortic stenosis
790D300	Creation of valved conduit between left ventricle and aorta	G132.13	Mitral regurgitation and aortic stenosis
790D400	Revision of valved cardiac conduit	G133.00	Mitral and aortic incompetence
790D600	Creation valved conduit between left vent heart pulm artery	G133.11	Mitral and aortic insufficiency
790D700	Replacement of valved cardiac conduit	G133.12	Mitral and aortic regurgitation
790Dy00	Other specified creation of valved cardiac conduit	G13y.00	Multiple mitral and aortic valve involvement
790Dz00	Creation of valved cardiac conduit NOS	G13z.00	Mitral and aortic valve disease NOS
790M300	Radical aortopulmonary recon rig vent pulmon art valvel cond	G140.00	Tricuspid valve disease NEC
791..00	Valves of heart and adjacent structures operations	G140000	Rheumatic tricuspid stenosis

7910.00	Plastic repair of mitral valve	G140100	Rheumatic tricuspid insufficiency
7910.11	Mitral valvuloplasty	G140111	Tricuspid regurgitation - rheumatic
7910.12	Replacement of mitral valve	G140112	Tricuspid incompetence - rheumatic
7910000	Allograft replacement of mitral valve	G140200	Rheumatic tricuspid stenosis and insufficiency
7910100	Xenograft replacement of mitral valve	G14021X	Rheumatic tricuspid stenosis and regurgitation
7910200	Prosthetic replacement of mitral valve	G14021Y	Rheumatic tricuspid stenosis and incompetence
7910211	Bjork-Shiley prosthetic replacement of mitral valve	G140300	Tricuspid stenosis, cause unspecified
7910212	Bjork-Shiley prosthetic replacement of mitral valve	G140400	Tricuspid insufficiency, cause unspecified
7910213	Carpentier prosthetic replacement of mitral valve	G140412	Tricuspid incompetence, cause unspecified
7910214	Edwards prosthetic replacement of mitral valve	G140413	Tricuspid regurgitation, cause unspecified
7910300	Replacement of mitral valve NEC	G140500	Tricuspid stenosis and insufficiency, cause unspecified
7910400	Mitral valvuloplasty NEC	G140511	Tricuspid stenosis and incompetence, cause unspecified
7910411	Mitral valve repair NEC	G140514	Tricuspid stenosis and regurgitation, cause unspecified
7910y00	Other specified plastic repair of mitral valve	G140z00	Rheumatic tricuspid valve disease NOS
7910z00	Plastic repair of mitral valve NOS	G141.00	Rheumatic pulmonary valve disease
7911.00	Plastic repair of aortic valve	G141100	Rheumatic pulmonary insufficiency
7911.11	Aortic valvuloplasty	G141200	Rheumatic pulmonary stenosis and insufficiency
7911.12	Replacement of aortic valve	G141z00	Rheumatic pulmonary valve disease NOS
7911000	Allograft replacement of aortic valve	G54..11	Heart valve disorders - non rheumatic
7911100	Xenograft replacement of aortic valve	G540.00	Mitral valve incompetence
7911200	Prosthetic replacement of aortic valve	G540.12	Mitral valve insufficiency

7911300	Replacement of aortic valve NEC	G540.14	Mitral valve regurgitation
7911400	Aortic valvuloplasty NEC	G540.15	Mitral valve prolapse
7911411	Aortic valve repair NEC	G540.16	Mitral regurgitation
7911500	Transapical aortic valve implantation	G540000	Mitral incompetence, non-rheumatic
7911600	Transluminal aortic valve implantation	G540100	Mitral incompetence, cause unspecified
7911y00	Other specified plastic repair of aortic valve	G540200	Mitral valve prolapse
7911z00	Plastic repair of aortic valve NOS	G540300	Mitral valve leaf prolapse
7912.00	Plastic repair of tricuspid valve	G540z00	Mitral valve disorders NOS
7912.11	Replacement of tricuspid valve	G541.00	Aortic valve disorders
7912.12	Tricuspid valvuloplasty	G541000	Aortic incompetence, non-rheumatic
7912000	Allograft replacement of tricuspid valve	G541011	Aortic insufficiency, non-rheumatic
7912100	Xenograft replacement of tricuspid valve	G541012	Aortic regurgitation, non-rheumatic
7912200	Prosthetic replacement of tricuspid valve	G541100	Aortic stenosis, non-rheumatic
7912300	Replacement of tricuspid valve NEC	G541200	Aortic incompetence alone, cause unspecified
7912400	Repositioning of tricuspid valve	G541211	Aortic insufficiency alone, cause unspecified
7912500	Tricuspid valvuloplasty NEC	G541212	Aortic regurgitation alone, cause unspecified
7912511	Tricuspid valve repair NEC	G541300	Aortic stenosis alone, cause unspecified
7912y00	Other specified plastic repair of tricuspid valve	G541400	Aortic valve stenosis with insufficiency
7912z00	Plastic repair of tricuspid valve NOS	G541500	Aortic stenosis
7913.00	Plastic repair of pulmonary valve	G541600	Aortic valve sclerosis
7913.11	Pulmonary valvuloplasty	G541700	Aortic valve calcification

7913.12	Replacement of pulmonary valve	G541z00	Aortic valve disorders NOS
7913000	Allograft replacement of pulmonary valve	G542.00	Tricuspid valve disorders, non-rheumatic
7913100	Xenograft replacement of pulmonary valve	G542000	Tricuspid incompetence, non-rheumatic
7913200	Prosthetic replacement of pulmonary valve	G542011	Tricuspid insufficiency, non-rheumatic
7913300	Replacement of pulmonary valve NEC	G542012	Tricuspid regurgitation, non-rheumatic
7913400	Pulmonary valvuloplasty NEC	G542100	Tricuspid stenosis, non-rheumatic
7913411	Pulmonary valve repair NEC	G542200	Nonrheumatic tricuspid valve stenosis with insufficiency
7913y00	Other specified plastic repair of pulmonary valve	G542X00	Nonrheumatic tricuspid valve disorder, unspecified
7913z00	Plastic repair of pulmonary valve NOS	G542z00	Tricuspid valve disorders NOS
7914.00	Plastic repair of unspecified valve of heart	G543.00	Pulmonary valve disorders
7914.11	Replacement of unspecified valve of heart	G543000	Pulmonary incompetence, non-rheumatic
7914000	Allograft replacement of valve of heart NEC	G543011	Pulmonary insufficiency, non-rheumatic
7914100	Xenograft replacement of valve of heart NEC	G543012	Pulmonary regurgitation, non-rheumatic
7914200	Prosthetic replacement of valve of heart NEC	G543100	Pulmonary stenosis, non-rheumatic
7914211	Edwards prosthetic replacement of valve of heart	G543200	Pulmonary incompetence, cause unspecified
7914212	Starr prosthetic replacement of valve of heart	G543213	Pulmonary insufficiency, cause unspecified
7914300	Replacement of valve of heart NEC	G543215	Pulmonary regurgitation, cause unspecified
7914400	Valvuloplasty of heart NEC	G543300	Pulmonary stenosis, cause unspecified
7914411	Repair of valve of heart NEC	G543311	Pulmonary stenosis, cause unspecified
7914500	Truncal valve repair	G543400	Pulmonary valve stenosis with insufficiency
7914600	Replacement of truncal valve	G543z00	Pulmonary valve disorders NOS

7914y00	Other specified plastic repair of unspecified valve of heart	G544.00	Multiple valve diseases
7914z00	Plastic repair of unspecified valve of heart NOS	G544000	Disorders of both aortic and tricuspid valves
7915.00	Revision of plastic repair of valve of heart	G544100	Disorders of both mitral and tricuspid valves
7915000	Revision of plastic repair of mitral valve	G544200	Combined disorders of mitral, aortic and tricuspid valves
7915100	Revision of plastic repair of aortic valve	G544X00	Multiple valve disease, unspecified
7915200	Revision of plastic repair of tricuspid valve	G54z.00	Endocarditis, valve unspecified
7915300	Revision of plastic repair of pulmonary valve	G54z000	Incompetence of unspecified heart valve
7915400	Revision of plastic repair of truncal valve	G54z013	Regurgitation of unspecified heart valve
7915y00	Other specified revision of plastic repair of valve of heart	G54z014	Insufficiency of unspecified heart valve
7915z00	Revision of plastic repair of valve of heart NOS	G54z100	Stenosis of unspecified heart valve
7916.00	Open incision of heart valve	G54z200	Chronic cardiac valvulitis NOS
7916000	Open mitral valvotomy	G54z300	Endocarditis, valve unspecified, OS
7916200	Open tricuspid valvotomy	G54z500	Valvular heart disease
7916y00	Other specified open specified incision of valve of heart	G54zz00	Endocarditis, valve unspecified, NOS
7916z00	Open incision of valve of heart NOS	G580400	Congestive heart failure due to valvular disease
7917.00	Closed incision of heart valve	Gyu1000	[X]Other mitral valve diseases
7917000	Closed mitral valvotomy	Gyu1100	[X]Other rheumatic aortic valve diseases
7917200	Closed tricuspid valvotomy	Gyu1200	[X]Other tricuspid valve diseases
7917311	Brock pulmonary valvulotomy	Gyu1300	[X]Other multiple valve diseases
7917y00	Other specified closed incision of valve of heart	Gyu1500	[X]Multiple valve disease, unspecified
7917z00	Closed incision of valve of heart NOS	Gyu5500	[X]Other nonrheumatic mitral valve disorders

7918.00	Other open operations on valve of heart	Gyu5600	[X]Other aortic valve disorders
7918000	Annuloplasty of mitral valve	Gyu5700	[X]Other nonrheumatic tricuspid valve disorders
7918100	Annuloplasty of tricuspid valve	Gyu5800	[X]Other pulmonary valve disorders
7918111	De Vega tricuspid annuloplasty	Gyu5900	[X]Mitral valve disorders in diseases classified elsewhere
7918200	Annuloplasty of valve of heart NEC	Gyu5A00	[X]Aortic valve disorders in diseases classified elsewhere
7918300	Excision of vegetations of valve of heart	Gyu5B00	[X]Tricuspid valve disorders/diseases CE
7918400	Closure of tricuspid valve	Gyu5C00	[X]Pulmonary valve disorders in diseases CE
7918500	Closure of pulmonary valve	Gyu5D00	[X]Multiple valve disorders/diseases CE
7918y00	Other specified other open operation on valve of heart	Gyu5E00	[X]Endocarditis, valve unspecified, in diseases CE
7918z00	Other open operation on valve of heart NOS	Gyu5f00	[X]Nonrheumatic tricuspid valve disorder, unspecified
7919.00	Therapeutic transluminal operations on valve of heart	P60..00	Pulmonary valve anomalies
7919000	Percutaneous transluminal mitral valvotomy	P600.00	Pulmonary valve anomaly, unspecified
7919200	Percutaneous transluminal tricuspid valvotomy	P601.00	Congenital atresia of the pulmonary valve
7919400	Percutaneous transluminal valvuloplasty	P601000	Hypoplasia of pulmonary valve
7919500	Percutaneous transluminal pulmonary valve perfor dilation	P601z00	Congenital atresia of pulmonary valve NOS
7919600	Percutaneous transluminal pulmonary valve replacement	P602100	Congenital fusion of pulmonary valve segment
7919y00	Therapeutic transluminal operation on heart valve OS	P60z.00	Other pulmonary valve anomalies
7919z00	Therapeutic transluminal operation on heart valve NOS	P60z000	Congenital insufficiency of the pulmonary valve
791A.00	Remove obstruction from structure adjacent to valve of heart	P60z200	Supernumerary pulmonary valve cusps
791A400	Excision of supramitral ring	P60zz00	Other pulmonary valve anomaly NOS
791Ay00	Removal of obstruction from struct adjacent heart valve OS	P61..00	Congenital tricuspid atresia and stenosis

791Az00	Removal of obstruction from struct adjacent heart valve NOS	P610.00	Congenital tricuspid atresia
791B.00	Other operations on structure adjacent to valve of heart	P611.00	Congenital tricuspid stenosis
791B200	Operations on mitral subvalvular apparatus	P61z.00	Congenital tricuspid atresia or stenosis NOS
791By00	Operation on structure adjacent to heart valve OS	P63..00	Congenital aortic valve stenosis
791Bz00	Operation on structure adjacent to valve of heart NOS	P64..00	Congenital aortic valve insufficiency
791C500	Aortoventriculoplasty with pulmonary valve autograft	P640.00	Congenital aortic valve insufficiency, unspecified
791D.00	Excision of valve of heart	P641.00	Bicuspid aortic valve
791D000	Tricuspid valvectomy	P64z.00	Congenital aortic valve insufficiency NOS
791D100	Pulmonary valvectomy	P65..00	Congenital mitral stenosis
791Dy00	Other specified excision of valve of heart	P650.00	Congenital mitral stenosis, unspecified
791Dz00	Excision of valve of heart NOS	P651.00	Fused commissure of the mitral valve
791y.00	Heart valve or adjacent structures operations OS	P652.00	Parachute deformity of the mitral valve
791z.00	Heart valve and adjacent structures operations NOS	P653.00	Supernumerary cusps of the mitral valve
7N40.00	[SO]Valve of heart	P65z.00	Congenital mitral stenosis NOS
7N40000	[SO]Mitral valve	P66..00	Congenital mitral insufficiency
7N40100	[SO]Aortic valve	P6W..00	Congenital malformation of aortic and mitral valves unsp
7N40200	[SO]Tricuspid valve	P6X..00	Congenital malformation of tricuspid valve, unspecified
7N40300	[SO]Pulmonary valve	P6yy.11	Hypoplastic aortic orifice or valve
7N40400	[SO]Truncal valve	P6yy.13	Congenital insufficiency of heart valve NEC
7N40y00	[SO]Specified valve of heart NEC	P6yy700	Atresia of heart valve NEC
7N40z00	[SO]Valve of heart NEC	P6yyB00	Supernumerary heart valve cusps NEC

A932.11	Syphilitic valve disease	P6yyC00	Fusion of mitral valve cusps
A932000	Syphilitic endocarditis of unspecified valve	P6yyD00	Fusion of heart valve cusps NEC
A932100	Syphilitic endocarditis of mitral valve	P6yyD11	Fusion of tricuspid valve cusps NEC
A932200	Syphilitic endocarditis of aortic valve	P6z0.00	Unspecified anomaly of heart valve
A932300	Syphilitic endocarditis of tricuspid valve	P722400	Supra-valvular aortic stenosis
A932400	Syphilitic endocarditis of pulmonary valve	Pyu2200	[X]Other congenital malformations of pulmonary valve
A932z00	Syphilitic endocarditis of heart valve NOS	Pyu2300	[X]Other congenital malformations of tricuspid valve
G11..00	Mitral valve diseases	Pyu2400	[X]Other congenital malformations of aortic & mitral valves
G11..11	Rheumatic mitral valve disease	Pyu2G00	[X]Congenital malformation of tricuspid valve, unspecified
G110.00	Mitral stenosis	Pyu2H00	[X]Congenital malformation of aortic and mitral valves unsp
G110.11	Rheumatic mitral stenosis	SP00200	Mechanical complication of heart valve prosthesis
G111.00	Rheumatic mitral insufficiency	SP00400	Infect and inflammatory reaction due to cardiac valve pros
G111.11	Mitral incompetence - rheumatic	SyuK611	[X] Embolism from prosthetic heart valve
G111.12	Mitral regurgitation - rheumatic	TB01200	Implant of heart valve prosthesis + complication, no blame
G112.00	Mitral stenosis with insufficiency	ZV42200	[V]Heart valve transplanted
G112.12	Mitral stenosis with incompetence	ZV43300	[V]Has artificial heart valve
G112.13	Mitral stenosis with regurgitation	ZV45H00	[V]Presence of prosthetic heart valve
G113.00	Nonrheumatic mitral valve stenosis	ZVu6e00	[X]Presence of other heart valve replacement
G114.00	Ruptured mitral valve cusp	P602.00	Congenital pulmonary stenosis
G11z.00	Mitral valve disease NOS	P602000	Congenital fusion of pulmonic cusps
G12..00	Rheumatic aortic valve disease	P62..00	Ebstein's anomaly

G121.00	Rheumatic aortic insufficiency	P65..11	Duroziez's disease
G121.12	Aortic regurgitation - rheumatic		

Chronic renal insufficiency

code	description	code	description
1Z10.00	Chronic kidney disease stage 1	1Z1D.00	Chronic kidney disease stage 3A with proteinuria
1Z11.00	Chronic kidney disease stage 2	1Z1D.11	CKD stage 3A with proteinuria
1Z12.00	Chronic kidney disease stage 3	1Z1E.00	Chronic kidney disease stage 3A without proteinuria
1Z13.00	Chronic kidney disease stage 4	1Z1E.11	CKD stage 3A without proteinuria
1Z14.00	Chronic kidney disease stage 5	1Z1F.00	Chronic kidney disease stage 3B with proteinuria
1Z15.00	Chronic kidney disease stage 3A	1Z1F.11	CKD stage 3B with proteinuria
1Z16.00	Chronic kidney disease stage 3B	1Z1G.00	Chronic kidney disease stage 3B without proteinuria
1Z17.00	Chronic kidney disease stage 1 with proteinuria	1Z1G.11	CKD stage 3B without proteinuria
1Z17.11	CKD stage 1 with proteinuria	1Z1H.00	Chronic kidney disease stage 4 with proteinuria
1Z18.00	Chronic kidney disease stage 1 without proteinuria	1Z1H.11	CKD stage 4 with proteinuria
1Z18.11	CKD stage 1 without proteinuria	1Z1J.00	Chronic kidney disease stage 4 without proteinuria
1Z19.00	Chronic kidney disease stage 2 with proteinuria	1Z1J.11	CKD stage 4 without proteinuria
1Z19.11	CKD stage 2 with proteinuria	1Z1K.00	Chronic kidney disease stage 5 with proteinuria
1Z1A.00	Chronic kidney disease stage 2 without proteinuria	1Z1K.11	CKD stage 5 with proteinuria
1Z1A.11	CKD stage 2 without proteinuria	1Z1L.00	Chronic kidney disease stage 5 without proteinuria
1Z1B.00	Chronic kidney disease stage 3 with proteinuria	1Z1L.11	CKD stage 5 without proteinuria

1Z1B.11	CKD stage 3 with proteinuria	K051.00	Chronic kidney disease stage 1
1Z1C.00	Chronic kidney disease stage 3 without proteinuria	K052.00	Chronic kidney disease stage 2
1Z1C.11	CKD stage 3 without proteinuria	K053.00	Chronic kidney disease stage 3
K055.00	Chronic kidney disease stage 5	K054.00	Chronic kidney disease stage 4

Stroke (not specified as haemorrhage or infarction)

**Code**	**Description**	**Code**	**Description**
G65z1	Intermittent CVA	G664.00	Cerebellar stroke syndrome
g66..	Stroke/CVA unspecified	G667.00	Left sided CVA
662e.00	Stroke/CVA annual review	G668.00	Right sided CVA
G61..11	CVA - cerebrovascular accid due to intracerebral haemorrhage	Gyu6C00	[X]Sequelae of stroke,not specified as haemorrhage or infarction
G66..11	CVA unspecified	1M4..00	Central post-stroke pain
G66..12	Stroke unspecified	G68X.00	Sequelae of stroke,not specified as haemorrhage or infarction
G66..13	CVA - Cerebrovascular accident unspecified	G66..00	Stroke and cerebrovascular accident unspecified
G663.00	Brain stem stroke syndrome		

Other pulmonary heart diseases

**Code**	**Description**	**Code**	**Description**
G41..00	Chronic pulmonary heart disease	Gyu4000	[X]Other specified pulmonary heart diseases

G410.00	Primary pulmonary hypertension	7Q01000	Primary pulmonary hypertension drugs band 1
G411.00	Kyphoscoliotic heart disease	7Q01100	Primary pulmonary hypertension drugs band 2
G41y.00	Other chronic pulmonary heart disease	7Q01200	Primary pulmonary hypertension drugs band 3
G41y000	Secondary pulmonary hypertension	7Q01300	Primary pulmonary hypertension drugs band 4
G41y100	Thromboembolic pulmonary hypertension	7Q01400	Pulmonary arterial hypertension drugs Band 1
G41yz00	Other chronic pulmonary heart disease NOS	7Q01500	Pulmonary arterial hypertension drugs Band 2
G41z.00	Chronic pulmonary heart disease NOS	7Q01600	Pulmonary arterial hypertension drugs Band 3
G41z.11	Chronic cor pulmonale	7Q01700	Pulmonary arterial hypertension drugs Band 4

Paroxysmal tachycardia

**Code**	**description**	**Code**	**Description**
3282.00	ECG: ventricular tachycardia	G572000	Essential paroxysmal tachycardia
G570.00	Paroxysmal supraventricular tachycardia	G572z00	Paroxysmal tachycardia NOS
G570000	Paroxysmal atrial tachycardia	G57y900	Supraventric. tachycardia NOS
G570100	Paroxysmal atrioventricular tachycardia	G57y700	Sinus tachycardia
G570200	Paroxysmal junctional tachycardia	G572z	Paroxysmal tachycardia NOS
G570300	Paroxysmal nodal tachycardia	3274.	ECG: paroxysmal atrial tachy.
G570z00	Paroxysmal supraventricular tachycardia NOS	G57yA	Re-entry ventricular arrhythmia
G571.00	Paroxysmal ventricular tachycardia	G5721	Bouveret-Hoffmann syndrome
G571.11	Ventricular tachycardia	G5720	Essential paroxysmal tachyc.
G572.00	Paroxysmal tachycardia unspecified	321C100	ECG: sinus tachycardia

Other cardiac arrhythmias

Code	Description	Code	Description
G57y.00	Other cardiac dysrhythmias	329..00	ECG: heart block
G57..00	Cardiac dysrhythmias	329Z.00	ECG: heart block NOS
G576.00	Ectopic beats	7L1H700	External ventricular defibrillation
G576200	Ventricular ectopic beats	G559.00	Arrhythmogenic right ventricular cardiomyopathy
G57y000	Persistent sinus bradycardia	G560.00	Complete atrioventricular block
G57y300	Sick sinus syndrome	G560.11	Third degree atrioventricular block
G576000	Ectopic beats unspecified	G561.00	Partial atrioventricular block
G577.00	Sinus arrhythmia	G561000	Atrioventricular block unspecified
G57z.00	Cardiac dysrhythmia NOS	G561100	First degree atrioventricular block
G576100	Supraventricular ectopic beats	G561200	Mobitz type II atrioventricular block
G574000	Ventricular fibrillation	G561300	Mobitz type I (Wenckebach) atrioventricular block
G57y100	Severe sinus bradycardia	G561311	Mobitz type 1 second degree atrioventricular block
G574.00	Ventricular fibrillat./flutter	G561400	Second degree atrioventricular block
G57y400	Sinoatrial node dysfunct. NOS	G561z00	Atrioventricular block NOS
G576z00	Ectopic beats NOS	G566000	Sinoatrial block
327..00	ECG: supraventricular arrhythmia	G566100	Interventricular block NOS
3262.00	ECG: extrasystole	G567.00	Anomalous atrioventricular excitation
G57yz00	Other cardiac dysrhythmia NOS	G567000	Accelerated atrioventricular conduction

G576300	Atrial premature depolarization	G567100	Accessory atrioventricular conduction
328..00	ECG: ventricular arrhythmia	G567200	Pre-excitation atrioventricular conduction
G57y600	Nodal rhythm disorder	G567300	Ventricular pre-excitation
G576500	Ventricular premat depolarization	G567z00	Anomalous atrioventricular excitation NOS
Gyu5a00	[X]Aortc valv diso/diseases CE	G56y100	Atrioventricular dissociation
3263.00	ECG: ventricular ectopics	G574011	Cardiac arrest-ventricular fibrillation
3264.00	ECG: atrial ectopics	G575.12	Asystole
326..00	ECG: ectopic beats	G576.11	Premature beats
G574100	Ventricular flutter	G576011	Extrasystoles
G576400	Junctional premat depolarizatn	G57y.12	Pulse missed beats
3283.00	ECG: ventricular fibrillation	G57y200	Brugada syndrome
G574z00	Ventric.fibrillat./flutter NOS	Gyu5U00	[X]Other and unspecified atrioventricular block
327Z.00	ECG: supraventric. arryth. NOS	Gyu5Z00	[X]Other and unspecified premature depolarization
G578.00	Atrial standstill	R002600	[D]Asystolic vasovagal syncope
326Z.00	ECG: ectopic beats NOS	R059.00	[D]Sinus bradycardia
328Z.00	ECG: ventricular arrythmia NOS	R05W.00	[D] Bradycardia, unspecified
3292.00	ECG: partial sinu-atrial block	Ryu0600	[X]Bradycardia, unspecified
3293.00	ECG:complete sinu-atrial block	SP02400	Mechanical complication-ventricular(CSF) communicating shunt
321C000	ECG: sinus bradycardia	U60C200	[X]Oth anti-dysrhythm drug caus advers eff therap use, NEC
ZV45M00	[V]Biventricular pacemaker in situ	ZS42800	Bradyarthria

Personal history of medical treatment

Code	Description	Code	Description
1....00	History / symptoms	661P.00	At inc risk bleed from aspirin
14…00	Past medical history	14ND.00	History of cholecystectomy
14Q..00	H/O: regular medication	14b0.00	H/O 1 hepatitis B vaccination
14N..00	H/O: surgery	14P9.00	H/O: tuberculosis drug therapy
114..00	Full history taken	1568.00	H/O: subcut contraceptiv usage
1561.00	H/O: oral contraceptive usage	1563.00	H/O: contraceptive cap usage
14Y0.00	Born by caesarean section	14P6.00	H/O: hormone replacement (HRT)
8BPF.00	Req lifelong warfarin therapy	14N5.00	H/O: pneumonectomy
14P3.00	H/O: insulin therapy	156D.00	H/O: postcoital contracep usag
112..00	Brief history taken	14N3.00	H/O: major orthopaedic surgery
14Z2.00	H/O: medical history NOS	159B.00	H/O: bilateral oophorectomy
1564.00	H/O: sheath usage	142Z.00	H/O: * NOS
11Z..00	History taken NOS	14N2.00	H/O: major abdominal surgery
1599.00	H/O: hysterectomy	ZV15700	[V]PH - Contraception
14P1.00	H/O: anticoagulant therapy	14P7.00	H/O: chemotherapy
14P2.00	H/O: steroid therapy	ZV1C000	[V]Per hst/lg-tm(c)use antcoag
1562	H/O: IUCD usage	14P4.00	H/O: anticonvulsant therapy
156E.00	H/O: intrauterine system usage	14O1.00	H/O: radiation exposure
14Z..00	H/O: medical history NOS	14N4100	H/O: lower limb amputation

14N3000	H/O Spinal surgery	14PZ.00	H/O: high risk medication NOS
14P..00	H/O: high risk medication	156A.00	H/O: transderm contracep usage
1561000	H/O: progestogen only OC usage	113..00	Intermediate history taken
14N7.00	H/O: splenectomy	14N1.00	H/O: major vascular surgery
14N9.00	H/O: R cataract extraction	8BPf.00	Req lifelong warfarin therapy
14NA.00	H/O: L cataract extraction	156B.00	H/O: withdrw contrac meth usag
14NC.00	H/O: Bilat cataract extraction	ZV1Cz00	[V]Per hist/medicl trtmnt,unsp
14N4.00	H/O: limb amputation	1562100	H/O: difficult IUCD fitting
156C.00	H/O: depot contracep inj usage	14N4000	H/O: upper limb amputation
14NZ.00	H/O: surgery NOS	ZV15100	[V]PH - Heart/great vessel op.
14V2.00	H/O: renal dialysis	ZVu6X00	[X]Pers hist/major surgery,NEC
15A9.00	H/O: myomectomy/hysterotomy	ZVu6.00	[X]Pers,hlth haz,fam/pers hist
14N6.00	H/O: cardiac surgery	ZV15300	[V]PH - Irradiation
14P5.00	H/O: immunosupressive therapy	ZV1C100	[V]Personal hist/rehab measure
14P8.00	H/O: prolongd cortsteroid thpy	ZVu6Y00	[X]Per hist/o medical treatmnt
11…00	Depth of history	1567	H/O: spermicid contracep usage
ZV1C.00	[V]Pers history/medical trtmnt	ZV15200	[V]PH - Other major organ op.
14N8.00	H/O: orchidectomy	ZV1Cy00	[V]Per hist/oth medical trtmnt
ZV1C200	[V]PH long term (curr) use war	1564000	H/O: female condom usage
ZVu6W00	[X]Per hst/l trm(cur)use/o med	14N4Z00	H/O: limb amputation NOS

[a]Read codes based on Hill, 2019[14]

S6 Table. Brier scores

	Brier score<br>Original intercept	Brier score<br>Recalibrated intercept
Belgium	0.0146	0.0107
Germany	0.0335	0.0335
Australia	0.0457	0.0457
France	0.0183	0.0107
UK	0.0311	0.0305

Supplemental Figures

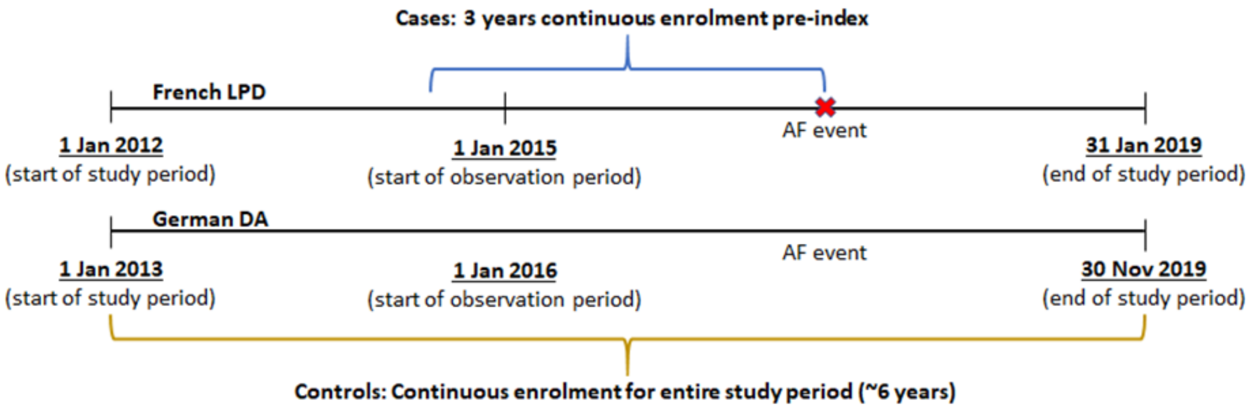

S1 Fig. Study design for the sensitivity analysis using recent data

S2 Fig. Correlation plots A) UK B) France C) Germany D) Belgium E) Australia

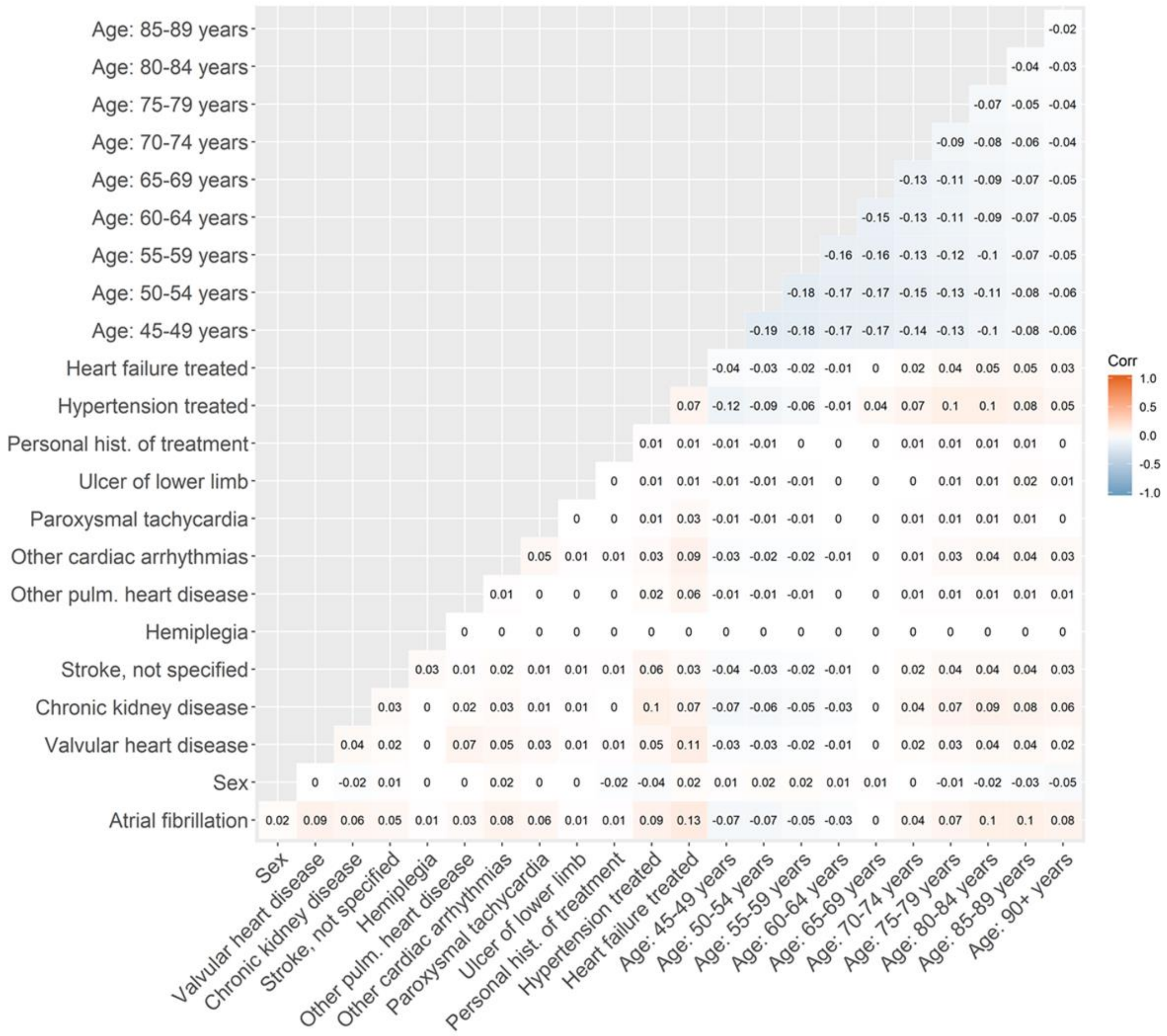

A

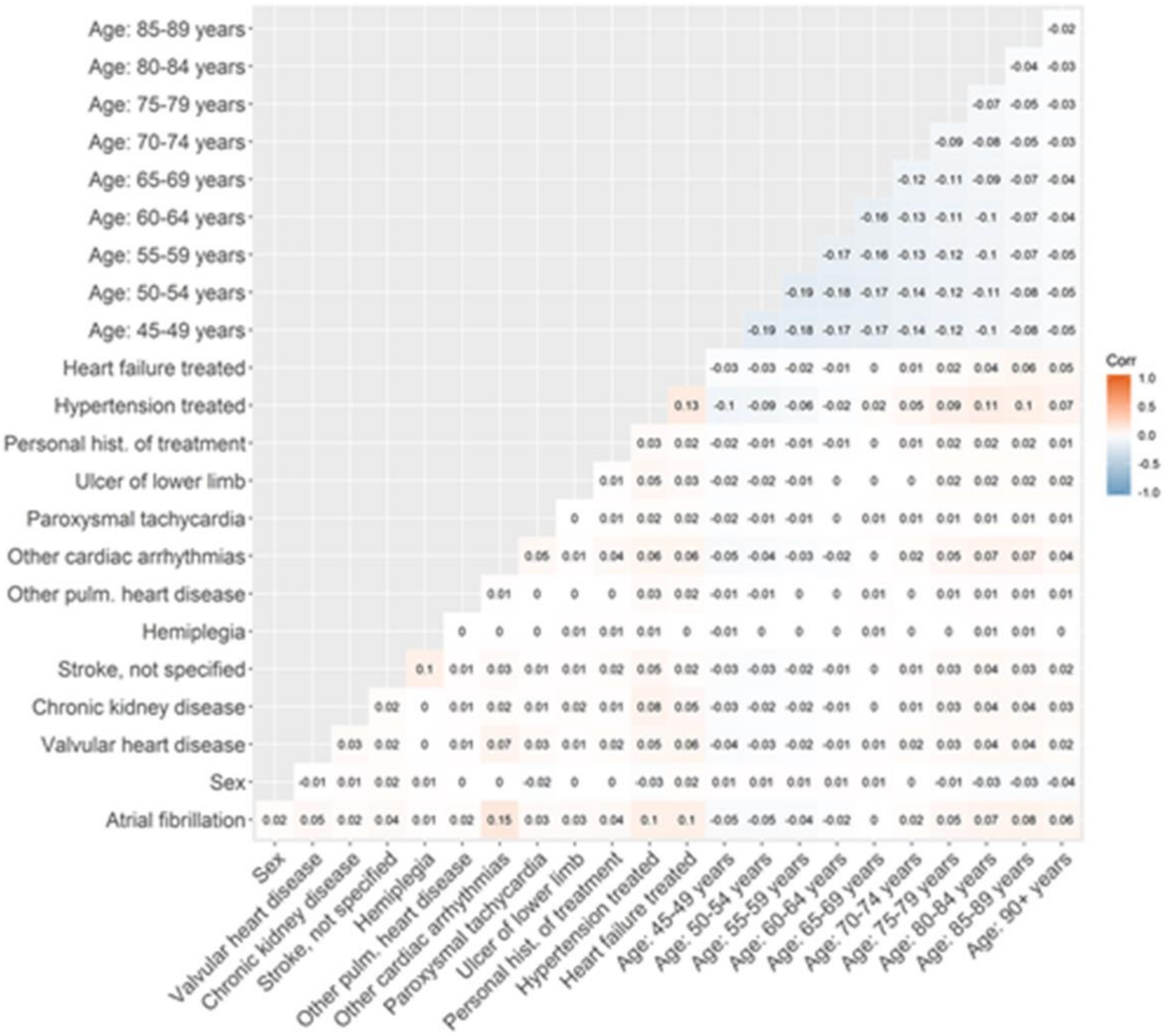
Age: 85-89 years
Age: 80-84 years
Age: 75-79 years
Age: 70-74 years
Age: 65-69 years
Age: 60-64 years
Age: 55-59 years
Age: 50-54 years
Age: 45-49 years
Heart failure treated
Hypertension treated
Personal hist. of treatment
Ulcer of lower limb
Paroxysmal tachycardia
Other cardiac arrhythmias
Other pulm. heart disease
Hemiplegia
Stroke, not specified
Chronic kidney disease
Valvular heart disease
Sex
Atrial fibrillation
Age: 90+ years
Corr
1.0
0.5
0.0
-0.5
-1.0

B

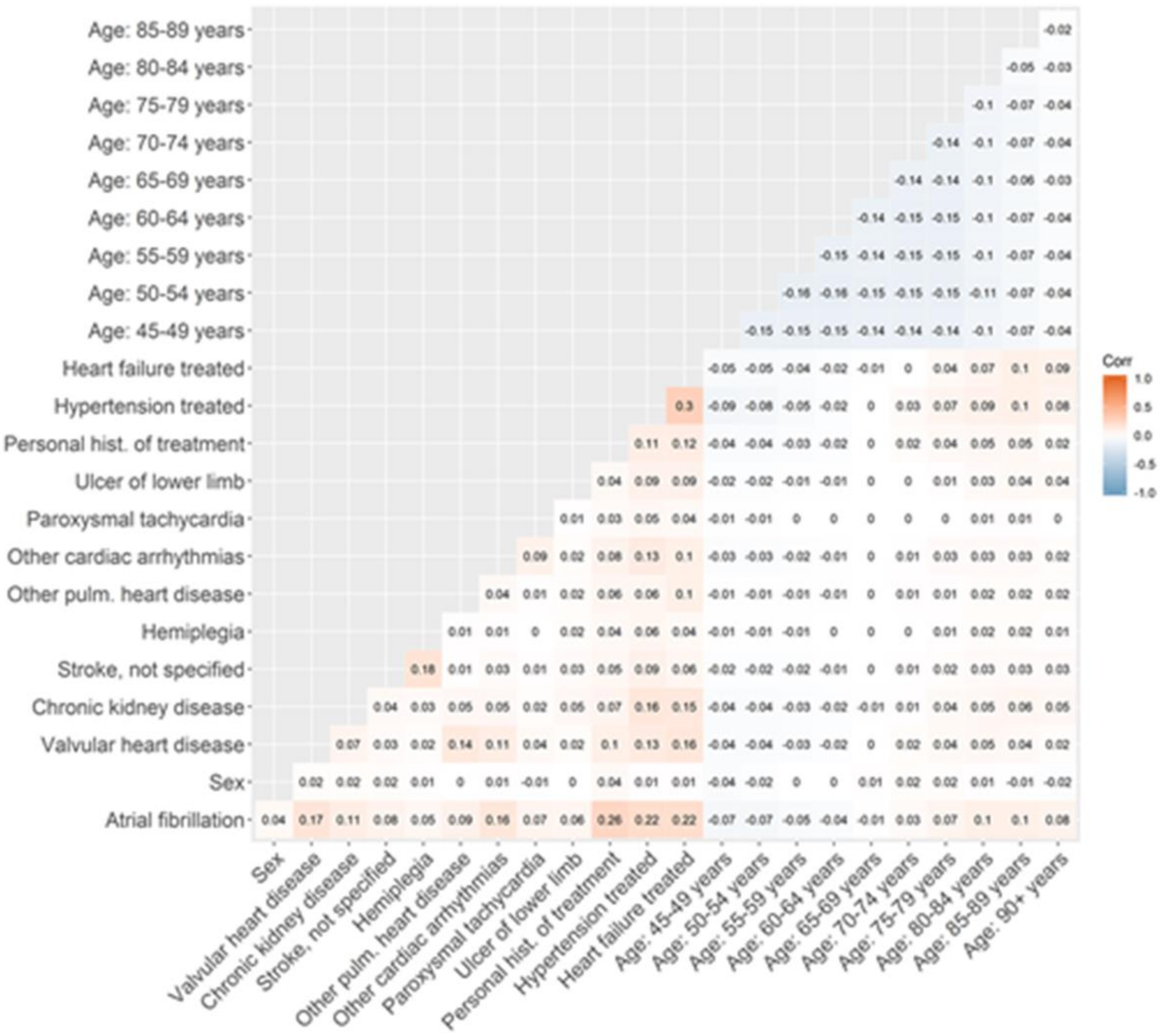
Age: 85-89 years
Age: 80-84 years
Age: 75-79 years
Age: 70-74 years
Age: 65-69 years
Age: 60-64 years
Age: 55-59 years
Age: 50-54 years
Age: 45-49 years
Heart failure treated
Hypertension treated
Personal hist. of treatment
Ulcer of lower limb
Paroxysmal tachycardia
Other cardiac arrhythmias
Other pulm. heart disease
Hemiplegia
Stroke, not specified
Chronic kidney disease
Valvular heart disease
Sex
Atrial fibrillation
Sex
Valvular heart disease
Chronic kidney disease
Stroke, not specified
Hemiplegia
Other pulm. heart disease
Other cardiac arrhythmias
Paroxysmal tachycardia
Ulcer of lower limb
Personal hist. of treatment
Hypertension treated
Heart failure treated
Age: 45-49 years
Age: 50-54 years
Age: 55-59 years
Age: 60-64 years
Age: 65-69 years
Age: 70-74 years
Age: 75-79 years
Age: 80-84 years
Age: 85-89 years
Age: 90+ years
Corr
1.0
0.5
0.0
-0.5
-1.0

C

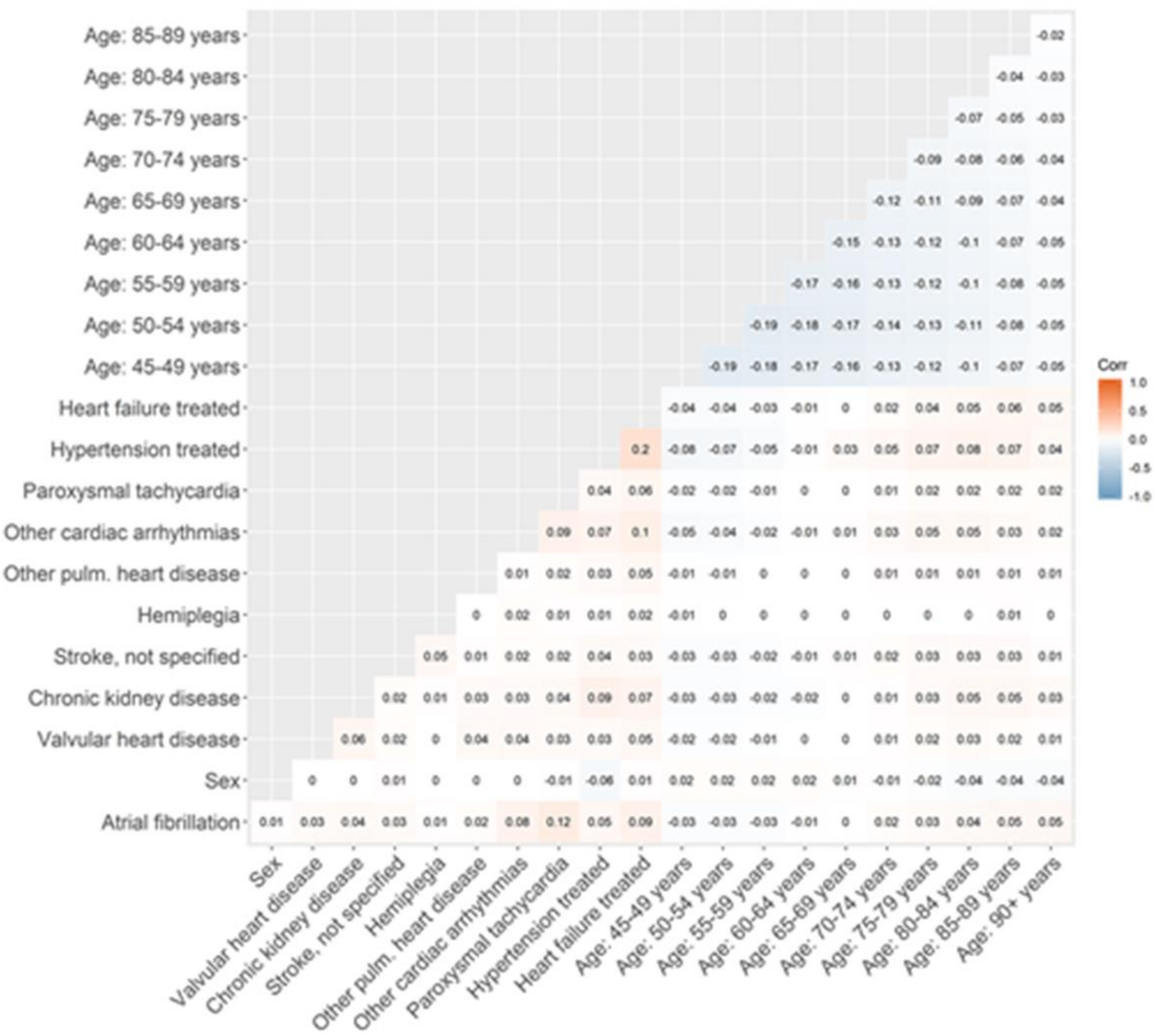
Age: 85-89 years
Age: 80-84 years
Age: 75-79 years
Age: 70-74 years
Age: 65-69 years
Age: 60-64 years
Age: 55-59 years
Age: 50-54 years
Age: 45-49 years
Heart failure treated
Hypertension treated
Paroxysmal tachycardia
Other cardiac arrhythmias
Other pulm. heart disease
Hemiplegia
Stroke, not specified
Chronic kidney disease
Valvular heart disease
Sex
Atrial fibrillation
Sex
Valvular heart disease
Chronic kidney disease
Stroke, not specified
Hemiplegia
Other pulm. heart disease
Other cardiac arrhythmias
Paroxysmal tachycardia
Hypertension treated
Heart failure treated
Age: 45-49 years
Age: 50-54 years
Age: 55-59 years
Age: 60-64 years
Age: 65-69 years
Age: 70-74 years
Age: 75-79 years
Age: 80-84 years
Age: 85-89 years
Age: 90+ years
Corr
1.0
0.5
0.0
-0.5
-1.0

D

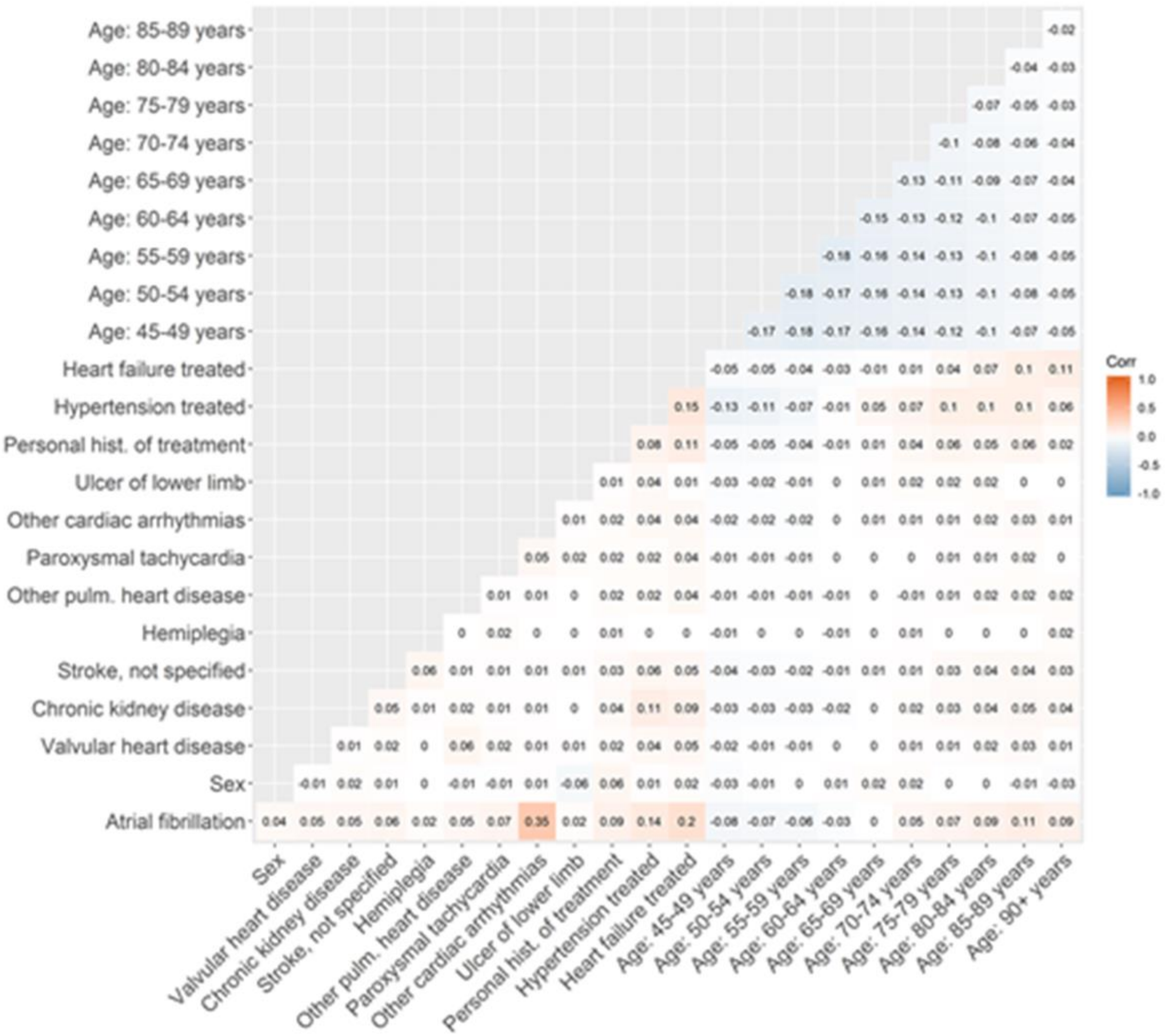
Age: 85-89 years
Age: 80-84 years
Age: 75-79 years
Age: 70-74 years
Age: 65-69 years
Age: 60-64 years
Age: 55-59 years
Age: 50-54 years
Age: 45-49 years
Heart failure treated
Hypertension treated
Personal hist. of treatment
Ulcer of lower limb
Other cardiac arrhythmias
Paroxysmal tachycardia
Other pulm. heart disease
Hemiplegia
Stroke, not specified
Chronic kidney disease
Valvular heart disease
Sex
Atrial fibrillation
Corr
1.0
0.5
0.0
-0.5
-1.0
Sex
Valvular heart disease
Chronic kidney disease
Stroke, not specified
Hemiplegia
Other pulm. heart disease
Paroxysmal tachycardia
Other cardiac arrhythmias
Ulcer of lower limb
Personal hist. of treatment
Hypertension treated
Heart failure treated
Age: 45-49 years
Age: 50-54 years
Age: 55-59 years
Age: 60-64 years
Age: 65-69 years
Age: 70-74 years
Age: 75-79 years
Age: 80-84 years
Age: 85-89 years
Age: 90+ years

E

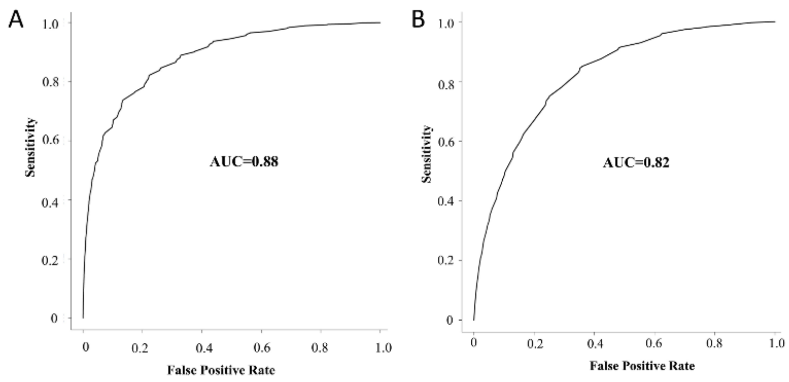

S3 Fig. Receiver operating characteristic (ROC) curves in the sensitivity analysis in A) Germany and B) France
